# The induced knockdown of GmCAD receptor protein encoding gene in Galleria mellonella decreased the insect susceptibility to a Photorhabdus akhurstii oral toxin

**DOI:** 10.1080/21505594.2021.2006996

**Published:** 2021-12-09

**Authors:** Tushar K. Dutta, Arudhimath Veeresh, Chetna Mathur, Victor Phani, Abhishek Mandal, Doddachowdappa Sagar, Suresh M. Nebapure

**Affiliations:** a Division of Nematology, ICAR-Indian Agricultural Research Institute, New Delhi, India; b Department of Agricultural Entomology, College of Agriculture, Uttar Banga Krishi Viswavidyalaya, Dakshin Dinajpur, India; c Division of Agricultural Chemicals, ICAR-Indian Agricultural Research Institute, New Delhi, India; d Division of Entomology, ICAR-Indian Agricultural Research Institute, New Delhi, India

**Keywords:** TcaB toxin, GmCAD receptor, *Photorhabdus* bacteria, *Galleria mellonella*, bioassay

## Abstract

*Photorhabdus* bacteria secrete a repertoire of protein toxins that can kill the host insect. Among them, toxin complex (Tc) proteins have gained significant attention due to their wider conservation across the different bacterial genera. In our laboratory, a C-terminal domain of TcaB protein was characterized from *P. akhurstii* bacterium that conferred the potent oral insecticidal effect on *Galleria mellonella*. However, the role of insect gut receptors in the TcaB intoxication process was yet to be investigated. In the current study, we examined the transcription of candidate midgut receptors in TcaB-infected larvae and subsequently cloned a cadherin-like gene, *GmCAD*, from *G. mellonella. GmCAD* was highly transcribed in the fourth-instar larval stage and specifically in the midgut tissues. Our ligand blot and binding ELISA assays indicated that TcaB binds to the truncated peptides from the GmCAD transmembrane-proximal region with greater affinity than that from the transmembrane-distal region. Oral administration of bacterially expressed *GmCAD* dsRNA in *G. mellonella* severely attenuated the expression of target mRNA, which in turn alleviated the negative effect of TcaB on insect survival (TcaB-induced mortality in CAD dsRNA pretreated larvae reduced by 72–83% compared to control), implying the association of GmCAD in the TcaB intoxication process. Present findings form a basis of future research related to the insect gut receptor interactions with *Photorhabdus* toxins.

## Introduction

Insect-parasitic nematodes from the families Heterorhabditidae and Steinernematidae have evolved a symbiotic relationship with the bacterial genera *Photorhabdus* and *Xenorhabdus*, respectively. The nematode–bacterium pair can kill the insect host (including Lepidoptera, Coleoptera, Diptera, Dictyoptera and Orthoptera orders) within 24–48 h through toxemia and septicemia [1,2]. Bacteria (reside in the nematode intestine) use its nematode partner for entry into the insect and nematode depends on bacteria for access of nutrients from the liquefied dead insect tissue. The bacteria use its repertoire of toxins and secondary metabolites, which actually kill the insect [3,4]. These nematodes have been extensively tested as insect biocontrol agents under laboratory conditions; however, their shorter shelf life and requirement of a narrow range of temperature and moisture for field efficacy have limited their commercial deployment [1].

A number of *Photorhabdus* toxins such as Tc, mcf, PirAB, Photox, Txp40, Pit, Galtox, RTX, and PVC were functionally characterized by investigating their biological activity in different insects [5–10]. To date, *Photorhabdus* Tc toxins have been extensively investigated [11,12] possibly because Tc displays oral insecticidal activity, which is amenable for its applicability via transgenic mean or bio-pesticide formulation alike *Bacillus thuringiensis* (Bt) Cry toxins. On the contrary, other *Photorhabdus* toxins are insecticidal per se by injection [13]. A number of Tc orthologues has been identified in other bacterial genera including *Xenorhabdus, Yersinia, Serratia, Pseudomonas, Burkholderia, Shewanella, Erwinia* and *Aeromonas*, suggesting that Tc gene is highly conserved in the Enterobacteriales order [6,14]. In addition, Tc orthologues were reported from gram-positive genera *Bacillus* [15].

In *P. luminescens*, tripartite Tc genes are grouped into three basic genetic elements, e.g. TcA, TcB, and TcC, which are found at four loci. *P. luminescens* strains TT01 and W14 contain a large variety of Tc genes with up to 7 TcA- and TcC-type genes [12]. TcA, TcB and TcC loci are similar to each other according to their encoded protein types, indicating the prevalence of Tc gene isoforms in the genome of *P. luminescens* [13]. Despite the promiscuity in Tc nomenclature, individual Tc components including Tca, Tcb, Tcc and Tcd independently conferred partial toxicity to various insects when expressed in *E. coli* [16–18]. *P. luminescens* TcdA expressed in *Arabidopsis thaliana* conferred insecticidal activity to *Manduca sexta* and *Diabrotica undecimpunctata* [19]. Toxin B (a 63 kDa protein) from *P. luminescens* W14 exhibited oral toxicity against *D. undecimpunctata* [20]. Conversely, it is suggested that Tc confers full toxicity when its individual components are co-expressed together. *P. luminescens* Tc toxin possesses its cytotoxic activity in the C-terminal hypervariable region of TcC. During the Tc intoxication process, this cytotoxic component is cleaved out from Tc holotoxin (TcB-TcC complex) and delivered into the host cell cytoplasm [11].

In our laboratory, a C-terminal domain of TcaB protein (63 kDa) was characterized from *P. akhurstii* bacterium (strains IARI-SGHR2 and IARI-SGMS1). TcaB has shown oral toxicity to the larvae of greater wax moth, *Galleria mellonella,* with LD_50_ values of 45.63–58.90 ng/g. When orally administered, TcaB had targeted the midgut epithelial cells and migrated to hemocoel by inducing leakiness in the basement membrane lining at midgut-hemocoel barrier. Next, a cytotoxic effect on hemocytes was documented, which was similar to apoptotic cell death. In parallel, TcaB caused an immunomodulatory effect by elevating the hemolymph phenoloxidase activity [21,22]. We identified a catalytic activity domain and a receptor binding domain in the TcaB sequence. Our *in silico* analysis suggested that TcaB putatively interacted with different insect gut receptors including cadherin (CAD), aminopeptidase N (APN), alkaline phosphatase (ALP), and ATP-binding cassette transporter subfamily C (ABCC) [22].

In the present study, we first cloned a CAD gene (*GmCad*) from *G. mellonella*. Heterologously expressed truncated CAD peptides (GmCADp1 and GmCADp2) could bind to *P. akhurstii* TcaB with varying potential. Oral delivery of bacterially expressed *GmCad* double-stranded RNA (dsRNA) considerably attenuated the target gene expression in *G. mellonella* that subsequently led to reduced susceptibility of insects to TcaB-induced larval mortality. Our results suggest that GmCAD may act as a transmembrane receptor during TcaB intoxication of *G. mellonella* gut epithelial cells.

## Materials and methods

### Rearing of insects and tissue collection

*G. mellonella* L. (Lepidoptera, Pyralidae) larvae were hatched from the eggs of a well-established laboratory population. Rearing of larvae was performed on the artificial diet consisting of twenty parts each of wheat and corn flour, two parts each of honey, milk powder and glycerol, and one part of yeast at 28°C and 70 ± 5% relative humidity (RH). Twenty mg ampicillin per kg of larval body mass was admixed to the diet to prevent any bacterial contamination. Larvae metamorphosed to the fourth-instar stage (0.45 ± 0.05 g body mass) were surface sterilized with cotton swabs dipped in 70% ethanol for further experimental use.

In order to investigate the stage and tissue-specific expression profiles of *GmCad* gene, whole bodies of different developmental stages of *G. mellonella* (first- to fifth-instar larvae) and different dissected body parts (head, fat body, foregut, midgut, and hindgut) were sampled independently by freezing quickly in liquid nitrogen and stored immediately at – 80°C for RNA extraction. Three biological replicates consisting of 15–20 larvae for each replicate were prepared for each treatment.

### RNA isolation, gene cloning, and bioinformatic analysis

Total RNA was extracted from the midgut tissue of fourth-instar larvae using TRIzol reagent (Invitrogen) by following the manufacturer’s protocol. Extracted RNA was digested with DNase I (TakaRa) to ward off genomic DNA contamination. RNA purity was determined in a Nanodrop ND-1000 spectrophotometer (Thermo Fisher Scientific), and integrity of RNA was assessed by resolving in 1% (w/v) agarose gel. One µg of total RNA was reverse transcribed to cDNA using first strand cDNA synthesis kit (Superscript VILO, Invitrogen) and preserved at – 20°C until further use.

According to the sequence of an uncharacterized *G. mellonella* cadherin-like protein (XP_026759573.1) in the NCBI non-redundant database, specific primers were designed. A Smart RACE (rapid amplification of cDNA ends) cDNA amplification kit (Clontech, TaKaRa) was used to synthesize 3ʹ and 5ʹ-RACE-ready cDNA from the first strand midgut cDNA and primed by oligo(dT) primer and Smart II A oligonucleotide by following the manufacturer’s protocol. 3ʹ- and 5ʹ-RACE fragments were generated by using sense and antisense gene-specific primers (GSP), respectively, accompanied by universal primers. Amplified products were cloned onto a pGEM-T Easy vector (Promega) and sequenced. After obtaining the complete cDNA, a set of primers was designed to verify the full-length sequence. The resulting sequence was named *GmCAD* and submitted to the NCBI GenBank repository. Primers were designed via Primer3Plus (http://www.bioinformatics.nl/cgi-bin/primer3plus/). Primer details are provided in Supplementary Table S1.

The full-length cDNA sequence was analyzed using an ORF finder tool (https://www.ncbi.nlm.nih.gov/orffinder/). The conserved domain structures in the sequence were examined via a NCBI conserved domain database (https://www.ncbi.nlm.nih.gov/Structure/cdd/) and a motif database search algorithm (https://www.genome.jp/tools/motif/). SignalP 5.0 (http://www.cbs.dtu.dk/) and TMpred (https://embnet.vital-it.ch/software/TMPRED) servers were used for prediction of signal peptide and transmembrane domain. O- and N-glycosylation sites were mined in NetOGlyc 4.0 (http://www.cbs.dtu.dk/services/NetOGlyc/) and NetNGlyc 1.0 (http://www.cbs.dtu.dk/services/NetNGlyc/) servers. Sequences were aligned with their homologues (identified via largest bit score and smallest expect value in NCBI BLASTp algorithm) in other insect species using the Clustal Omega multiple sequence alignment tool (https://www.ebi.ac.uk/Tools/msa/clustalo/). A phylogenetic tree was constructed using the MEGA X bioinformatics tool. The evolutionary history was predicted by a maximum likelihood method involving the Le and Gascuel model and selection via MODELTEST. Bootstrap consensus was generated from 1000 replicates, and branches corresponding to <70% replicates were collapsed. Gamma distribution was employed to model the evolutionary rate differences between sites [5 categories (+*G*, parameter = 2.3676)]. The initial tree for heuristic search was obtained by adopting the neighbor-joining method; the JTT model was used for pairwise distance estimation, and based on the log likelihood value, topology was selected.

Protein three-dimensional structures were modeled using homology modeling in SWISS-MODEL server. For accurate sequence alignment, the resulting model was adjusted manually using the graphics program in Discovery Studio v. 2.5.5 (Biovia). Protein–protein docking was performed using the same software with previously described adjustments [7,22–24].

### RT-qPCR assay

RNA was extracted from different body parts and developmental stages of *G. mellonella* (Supplementary Figure S1) and converted to cDNA as explained above. To analyze the stage- and tissue-specific expression of *GmCAD* gene, RT-qPCR was carried out in a Realplex^2^ thermal cycler (Eppendorf). A 10 μL reaction mixture for each sample consisted of 1.5 ng cDNA, 750 nM of sense and antisense primer and 5 μL SYBR Green PCR master-mix (Eurogentec). qPCR reaction conditions were a hot start of 95°C for 30 s, followed by 40 cycles of 95°C for 10 s and 60°C for 30 s. For assessing the amplification specificity, a melt curve program (95°C for 15 s, 60°C for 15 s, followed by a slow ramp from 60 to 95°C) was used. Quantification cycle (Cq) values were obtained from Realplex^2^ software (Eppendorf). Housekeeping genes of *G. mellonella*, i.e. *18S rRNA* and *EF-1α* (elongation factor) [25,26], were used as the internal reference. Fold change in target gene expression was determined using the 2^−ΔΔCq^ method. Five biological and three technical replicates were performed for each of the samples. RT-qPCR primers were designed using the OligoAnalyzer tool (https://eu.idtdna.com/). To estimate the reaction efficiency of RT-qPCR primers, a five-fold dilution series of fourth-instar larval cDNA (reverse-transcribed from 1 µg RNA) was used to generate the standard curve (Cq value versus cDNA concentration) followed by calculation of efficiency from the slope using linear regression by following the equation: *E* = (10^(−1/slope)^ – 1) × 100. Primer detail and reaction efficiency are provided in Supplementary Table S2.

### In vitro *production of TcaB and GmCAD protein fragments*

A stock culture of *E. coli* strain BL21 [DE3) containing the recombinant pET29a::*TcaB* expression clone was maintained in our laboratory. The methodology for TcaB cloning, expression, and purification are detailed in 21 and 22. Briefly, the recombinant *E. coli* cells were cultured in LB medium containing kanamycin (50 µg mL^−1^) at 37°C for 4 h or until the absorbance reached 0.6 at 600 nm. 1 mM isopropyl-β-D-thiogalactopyranoside (IPTG) was added in the medium to induce TcaB expression. *E. coli* cells expressing TcaB were harvested by centrifugation (8000 g for 20 min at 4°C) and lysed by sonication in an isolation buffer (2 M urea, 0.5 M NaCl, 20 mM Tris-HCl, 2% Triton X-100, pH 7). TcaB inclusion bodies were extracted from the crude cell lysate by centrifugation (12,000 g for 15 min at 4°C) and solubilized in a binding buffer (8 M urea, 0.5 M NaCl, 20 mM Tris-HCl, 5 mM imidazole, pH 7) by constant stirring at 28°C for 1 h. TcaB with His tag was purified by a nickel–nitrilotriacetic acid (Ni-NTA) affinity column (Qiagen) and eluted in 500 mM imidazole. His tag was digesteded by adding enterokinase, and the cleaved protein was refolded by following the manufacturer’s (Qiagen) protocol. Purified TcaB was dissolved in phosphate buffered saline (PBS, pH 7.0), and its concentration was determined by Bradford’s method using bovine serum albumin (BSA) as the standard protein. Protein identity was ascertained by resolving the sample in 12% SDS-PAGE followed by mass-spectrometry of the in-gel tryptic digests.

**Figure 1.**
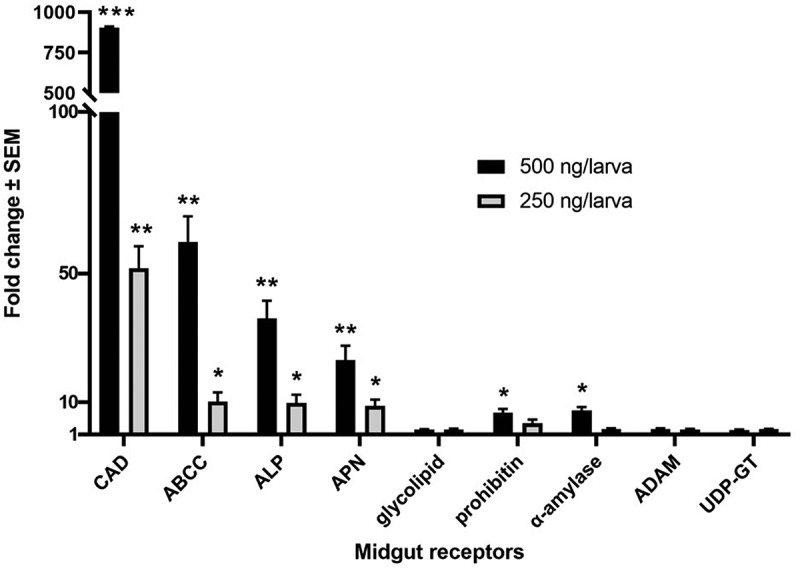
**Expression of putative Bt receptor genes in the midgut of *Galleria mellonella* larvae at 6 h after ingestion of TcaB toxin**. Lethal (500 ng/larva) and sub-lethal (250 ng/larva) concentrations of TcaB were used. Asterisks (*p < 0.05, **p < 0.01, ***p < 0.001) indicate significant differential fold change of candidate genes compared to their baseline expression (fold change value was set at 1) in larvae at 6 h after ingestion of PBS, Tukey’s HSD test. Gene expression was normalized using *G. mellonella 18S rRNA* and *EF-1α* genes. Each bar represents the mean fold change value with standard error of RT-qPCR runs in three biological (consisting of 15–20 larvae) and three technical replicates

**Figure 2.**
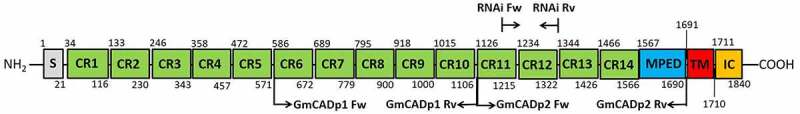
**The domain structure of GmCAD from *G. mellonella***. Gray box indicates N-terminal signal peptide (s). Characteristic cadherin repeats (CR1-14) are indicated by green boxes. The membrane-proximal extracellular domain (MPED), transmembrane domain (TM), and internal cytoplasmic domain (IC) are shown by blue, red, and orange boxes, respectively. Relative amino acid (aa) positions of different domains are shown. Arrows indicate the positions of primers to generate peptide I (GmCADp1: CR6-CR10, 572–1125 aa) and peptide II (GmCADp2: CR11-MPED, 1126–1690 aa) for ligand binding assay and dsRNA sequence (1187–1329 aa) for RNAi assay

In order to determine the potential TcaB-binding regions in GmCAD, we used two pairs of primers (GmCADp1Fw and GmCADp1Rv; GmCADp2Fw and GmCADp2Rv) to PCR-amplify the partial GmCAD fragments corresponding to bases 1716–3375 and 3378–5070 in the *GmCad* coding sequence. These *GmCAD* cDNA fragments correspond to amino acid residues 572–1125 from cadherin repeat 6 to 10 (CR6-CR10: GmCADp1) and 1126–1690 from cadherin repeat 6 to membrane-proximal extracellular domain (CR11-MPED: GmCADp2), respectively (Figure 1 and 2). *GmCADp1* and *GmCADp2* fragments were PCR-amplified from the first strand midgut cDNA using high-fidelity Phusion DNA polymerase (Invitrogen) using sense and antisense primers containing *Bam*HI and *Hind*III endonuclease sites at the 5ʹ ends, respectively (Supplementary Table S2). Gel-purified PCR products were double-digested with *Bam*HI and *Hind*III (New England Biolabs) at 37°C for 10 min and ligated into the previously digested pET29a vector (Invitrogen) using T4 DNA ligase (Promega) to generate pET29a::*GmCADp1* and pET29a::*GmCADp2* plasmids. The coding sequences and construct orientations were ascertained by sequencing. *E. coli* BL21 (DE3) cells were transformed with recombinant plasmids by electroporation and positive transformants were selected that exhibited resistance to kanamycin (50 µg mL^−1^) in LB medium. Protein expression, purification and identity confirmation was performed as described above.

### Ligand blot assay

Purified TcaB was biotin labeled using a EZ-Link sulfo-*N*-hydroxysuccinimide (NHS) liquid chromatography (LC) biotinylation kit (Thermo Fisher Scientific) by following the manufacturer’s protocol. Next, 5 µg of purified GmCAD fragments dissolved in loading buffer were heat-denatured and separated in 4–20% gradient SDS-PAGE gel (BioRad) and electro-transferred onto a polyvinylidene fluoride (PVDF) membrane (Millipore). The transblotted membrane was blocked overnight in PBST (PBS containing 0.05% Tween-20) and incubated with biotinylated TcaB (5 µg mL^−1^) in blocking buffer for 1 h at 28°C. After three washes (10 min each) with PBST, the membrane was probed with horseradish peroxidase (HRP)-conjugated anti-rabbit IgG (Sigma-Aldrich) in blocking buffer (1: 10,000 dilution) for 1 h at 28°C. After three washes, the membrane was developed using a ECL chemiluminescence western blotting kit (BioRad).

### Binding ELISA

The binding of TcaB to the GmCAD fragments was investigated by enzyme-linked immunosorbent assay (ELISA). Individual wells of 96-well plates (Costar 9018, Sigma-Aldrich) were coated with purified GmCAD (1 µg in 100 µl PBS) at 4°C overnight. Next, each well of the plate was washed thrice (10 min each) with 200 µl PBST to remove unbound protein followed by blocking each well using 200 µl blocking buffer (PBST containing 1% BSA) at 28°C by motorized shaking (80 rpm). Subsequently, each well was washed thrice (10 min each) with 200 µl PBST followed by addition of biotinylated TcaB protein (at different concentrations in 100 µl blocking buffer) to each well and incubated at 28°C for 1 h by shaking (80 rpm). Post incubation, unbound proteins were removed by washing as described above and wells were incubated with HRP-conjugated streptavidin (Sigma-Aldrich) in blocking buffer (1: 10,000 dilution) at 28°C for 1 h. After subsequent washes, 100 µl of 3,3ʹ,5,5ʹ-tetramethylbenzidine (TMB) ELISA substrate (fresh prepared) was added to each well and incubated at 28°C for 30 min. The reaction was terminated by adding 50 µl of 0.5 M H_2_SO_4_ in each well, and optical density (OD_450_) values were determined using a microplate reader (BioTek). The specific GmCAD-TcaB binding potential was estimated by subtracting the nonspecific binding (correspond to the presence of excess unlabeled TcaB protein) from the total binding potential. Data were analyzed using GraphPad Prism v.9.0.0 (GraphPad software Inc.).

### *Extraction of* G. mellonella *midgut juice and evaluation of its effect on purified TcaB*

The gut juice was extracted on ice from the dissected midgut of fourth-instar larvae (midgut tissues were carefully separated from food bolus containing peritrophic membrane). Ten samples were pooled together; the content was homogenized using 2 ml of ice-cold 0.15 M NaCl and centrifuged at 10,000g for 10 min at 4°C to obtain the clear supernatant. The protein concentration of gut juice was determined by Bradford’s method. Five µg each of TcaB separately mixed with the gut juice in four different concentrations (5, 10, 20, and 40 µg) in a final volume of 20 µl of Na_2_CO_3_ buffer (100 mM, pH 10.5) and incubated at 37°C for 1 h. The reaction was terminated by adding 1 µl of 10 mM phenylmethylsulfonyl fluoride (Sigma-Aldrich). Samples were separated in 12% SDS-PAGE and transblotted onto a PVDF membrane for Western blot detection. The membrane was blocked overnight in PBST and incubated with an anti-TcaB antibody (1: 10,000 dilution) in PBS for 1 h at 28°C. Subsequently, the membrane was incubated with HRP-conjugated rabbit antibody and the blot was developed as described above.

### DsRNA preparation

The region for RNA silencing in *GmCAD* was determined by analyzing the coding sequence in multiple dsRNA/siRNA designing tools including dsCheck (http://dscheck.rnai.jp/), Dharmacon (http://horizondiscovery.com/), and siDirect (http://sidirect2.rnai.jp/). We used L4440 plasmid (Addgene; contains two T7 promoters in inverted orientation flanking the multiple cloning site) to produce *GmCAD* dsRNA. A 427 bp fragment of *GmCad* gene corresponding to 1187–1329 aa of GmCAD protein was PCR amplified from larval midgut cDNA using specific primers containing *Sac*I and *Hind*III endonuclease sites (Supplementary Table S2). The product was ligated into *Sac*I and *Hind*III-digested plasmid L4440 to generate recombinant clones. *E.coli* HT115(DE3) competent cells (RNase III deficient) were prepared by the standard CaCl_2_ method and transformed with recombinant L4440. Individual colonies of HT115 cells were grown in LB medium supplemented with 50 µg mL^−1^ ampicillin and 12.5 µg mL^−1^ tetracycline at 37°C overnight with shaking (200 rpm). Induction of T7 polymerase synthesis was performed by adding 0.4 mM IPTG, and bacterial cells were incubated for an additional 4 h at 37°C. The expressed dsRNA was isolated from aliquots of bacteria and checked by electrophoresis on 1% (w/v) agarose gel (Supplementary Figure S2). Bacteria were precipitated by centrifugation at 5000 g for 10 min, re-suspended in 0.05 M PBS at 10: 1 ratio, and used for bioassay. The recombinant L4440 plasmid containing a *gfp* gene (Genbank ID: HF675000) was used to synthesize the control GFP dsRNA.

### RNAi bioassay

Our preliminary investigations showed that ingestion of dsRNA-expressing bacteria could liberate intact dsRNAs in a larval gut. A force feeding-based oral delivery [22] was performed to examine the effect of *GmCAD* dsRNA on *G. mellonella* larval sensitivity to TcaB toxin. First, 20 µL solution of HT115 clone expressing *GmCAD* dsRNA or *gfp* dsRNA (correspond to ~ 10 µg dsRNA according to our preliminary investigation) or 0.05 M PBS (negative control) was orally injected to a 12 h starved fourth-instar larvae using a sterilized 26-gauge hypodermic needle (Hamilton syringe, Sigma-Aldrich). Individual larvae were placed in sterile 6-well tissue culture plates containing artificial diet and incubated at 28°C in dark. At 24 h after dsRNA treatment, larvae were force-fed with 20 µL PBS containing TcaB (in different doses) using the sterilized needle; negative control consisted of PBS only. Larvae were incubated on artificial diet in 6-well plates at 28°C. After another 24 h insect mortality data was recorded. The experiment was replicated 5 times using a total of 150 larvae for each treatment.

RNAi knockdown of *GmCAD* was verified by RT-qPCR. RNA was extracted from the midgut samples of ten larvae from each dsRNA treatment group with three replicated samples and converted to cDNA as described above. RT-qPCR reaction conditions were followed as described above. Primer details and efficiency are provided in Supplementary Table S2.

### Data analysis

Gene expression and insect mortality data were subjected to normality test using Shapiro–Wilk and Kolmogorov–Smirnov test followed by one-way or two-way ANOVA with Tukey’s honest significant difference (HSD) test for multiple comparison in GraphPad Prism v.9.0.0 (GraphPad software Inc.).

## Results

### *Temporal expression profile of putative Bt toxin receptors in* G. mellonella *after TcaB ingestion*

Using reported Bt receptor sequences (CAD, ABCC, ALP, APN, glycolipid, prohibitin, α-amylase, ADAM metalloprotease and UDP-glucosyltransferase) from other lepidopteran insects as a query in local BLAST (E value cut off <1.0E^−30^), a number of corresponding homologues (having maximum query coverage and highest bit score) were identified from *G. mellonella* transcriptome data (NCBI BioProject ID: PRJNA498111). RT-qPCR primers for each receptor genes were designed based on the corresponding sequences retrieved from the local BLAST. We observed the significant differential expression of a number of these receptor mRNAs in the midgut tissue of *G. mellonella* fourth-instar larvae at 6 h after ingestion of lethal (500 ng/larva) and sub-lethal (250 ng/larva) concentrations of TcaB [a LD_90_ value of 377.4 ng/larva at 24 h post inoculation was obtained in our earlier study [22], compared to control (larvae force fed with PBS)]. Specifically, expression of CAD was increased by more than 900- (p < 0.001) and 50-folds (p < 0.01), in insects treated with lethal and sublethal doses of the toxin, respectively. In addition, ABCC, ALP and APN were highly expressed in midgut tissues exposed to all the toxin doses. Interestingly, increased transcription of prohibitin and α-amylase (p < 0.05) was documented in insects treated with lethal dose of the toxin (Figure 1). The higher induction of CAD mRNA upon lethal dose (500 ng/larva) of TcaB force feeding may be explained by the possibility of *de novo* synthesis of CAD gene at the midgut epithelial cell membrane in order to compensate for CAD deficiencies (arising due to their greater binding affinity with TcaB protein) during the initial 6 h critical period. The excess variation in CAD expression suggests that CAD maybe a virulence determinant of TcaB toxin. Differences in the temporal expression of receptor genes in insects treated with lethal and sub-lethal TcaB doses likely reflect the dose-dependent differences in the translation of receptor mRNAs. Increased transcription of prohibitin and alpha-amylase in larval midgut exposed to lethal TcaB dose is probably because both prohibitin and α-amylase may interact with TcaB at the extracellular space of gut epithelium. Furthermore, a protein-protein docking analysis was performed which showed that prohibitin and α-amylase dock with TcaB at multiple amino acid positions with greater binding energy potential (Supplementary Figure S3).

### *Cloning, sequence analysis and phylogeny of* GmCAD

Using 3ʹ- and 5ʹ-RACE and primer walking, a single cDNA containing the entire coding sequence of *GmCAD* was obtained (Supplementary Table S1). *GmCAD* cDNA (obtained NCBI Genbank accession number: MW355654) consists of a 5ʹ untranslated region, an open reading frame (ORF) and a 3ʹ untranslated region. *GmCAD* ORF (5,523 bp) encodes 1840 amino acids (aa) and shares a high degree of nucleotide identity (97.94%) with a cadherin-like cDNA from *G. mellonella* (XP_026759573.1). We considered these two sequences as allelic variants. The predicted GmCAD protein sequence has a calculated molecular mass of 200,742 Da and an isoelectric point of 4.46.

**Figure 3.**
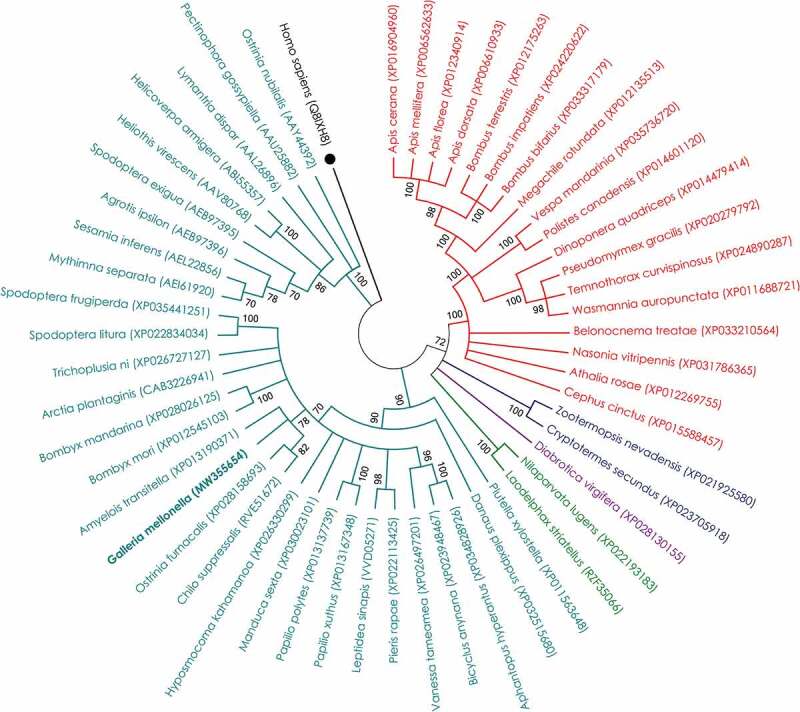
**Evolutionary relationship of CAD protein from *G. mellonella* with corresponding homologs from other insects**. The evolutionary history was inferred by Maximum Likelihood method based on Le and Gascuel model. Bootstrap consensus was inferred from 1000 replicates and branches corresponding to less than 70% replicates were collapsed. The analysis involved 54 amino acid sequences; all gaps and missing data positions were eliminated, and a total of 623 positions remained in the final dataset. NCBI Genbank accession numbers of different entries are provided in parentheses. The corresponding sequence from *Homo sapiens* was used as out-group (marked with ●), and entry for *G. mellonella* was kept in bold font. Entries in red, navy blue, purple, green, and teal colors correspond to the representative members of orders Hymenoptera, Isoptera, Coleoptera, Hemiptera, and Lepidoptera, respectively. Evolutionary analyses were conducted in MEGA X software

GmCAD consists of a signal peptide, 14 cadherin repeat domains, a membrane-proximal extracellular domain, a transmembrane domain and an internal cytoplasmic domain (Figure 2; Supplementary Figure S4). Thirty-four putative calcium-binding sites (DXNDN/DXD/ LDRE/QAV/HAV) are distributed throughout the extracellular domain (Supplementary Figure S4). Putative O-glycosylated serine/threonine residues and N-glycosylated asparagine residues were identified at numerous positions in the predicted protein (Supplementary Figure S4). A maximum likelihood method-based phylogenetic tree was constructed for comparing the highly homologous (50–83% amino acid identity (Query coverage: 97–100%; E value: 0) with GmCAD) cadherin-like protein sequences across the insect orders including Lepidoptera, Hemiptera, Coleoptera, Isoptera and Hymenoptera; an orthologous *Homo sapiens* cadherin was used as the outgroup. GmCAD is closely related to lepidopteran CADs from *Amyelois transitella, Bombyx mori, B. mandarina, Arctia plantaginis, Trichoplusia ni, Spodoptera litura, S. frugiperda, Ostrinia furnacalis, Chilo suppressalis, Hyposmocoma kahamanoa, Manduca sexta, Papilio polytes, P. xuthus* etc. Interestingly, lepidopteran CAD sequences are highly conserved as corresponding sequences from Hymenoptera, Isoptera, Coleoptera and Hemiptera branches away from the Lepidoptera group (Figure 3).

### *Stage- and tissue-specific expression profiles of* GmCAD

**Figure 4.**
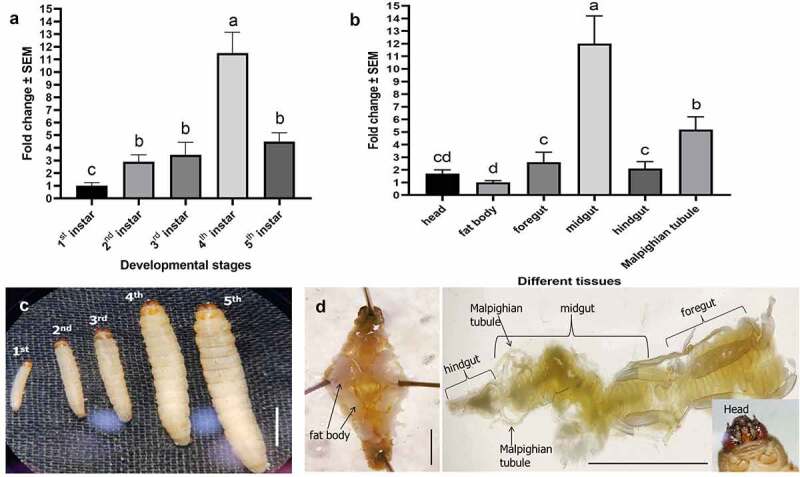
**Expression of *GmCAD* mRNA in different developmental stages** (a) **and in different tissues of fourth-instar stage** (b) **of *G. mellonella***. Relative to the fold change in first-instar stage (for stage-specific expression) and fat body (for tissue-specific expression), expression levels were determined in different treatments. Different letters indicate significant differential expression at p < 0.01, Tukey’s HSD test. Gene expression was normalized using *G. mellonella 18S rRNA* and *EF-1α* genes. Each bar represents the mean fold change value with standard error of RT-qPCR runs in three biological (consisting of 15–20 larvae) and three technical replicates. The photomicrographs of different larval stages (c) and dissected tissues (d) are provided in the bottom panels. Scale bar = 0.5 cm

Expression of *GmCAD* transcripts was analyzed by RT-qPCR in different developmental stages and tissues of fourth-instar larvae of *G. mellonella. GmCAD* was expressed in all the larval stages and greatest (p < 0.01) expression was detected in the fourth-instar. *GmCAD* was abundantly (p < 0.01) transcribed in the midgut tissues compared to its lower levels of expression in foregut, hindgut, head, fat body and Malpighian tubules (Figure 4).

### TcaB is highly homologous to specific bacterial toxins

Earlier, using a domain conservation analysis via multiple threading and segment assembly, we showed that *P. akhurstii* TcaB is highly homologous to *Bacillus* sp. crystal protein (PDB accession number: 1J0M), *Bacillus thuringiensis* Cry2Aa toxin (1I5P), *Yersinia entomophaga* Tc toxin (6OGD), and *P. luminescens* TcdA toxin (4O9Y) [22]. Protein homology based on the protein secondary/tertiary structure similarity often provides more valuable information about the protein function than amino acid sequence similarity-based analyses because potential structure conservation may be unraveled in addition to the sequence similarity information. Herein, we performed the secondary structure alignment of TcaB with above-mentioned proteins using the jFAT-CAT algorithm. Aligned structures used a fast algorithm to determine the initial seed alignment based on a hash table and subsequently expanded the seed alignment into the full alignment. The similarity of a protein pair was calculated based on the coordinates of their Cα atoms. A global optimization was performed in which a large number of combinations of residue equivalence in three-dimensional space was searched to obtain an optimal structure alignment. According to the greater % structural similarity (based on overlapped residues) and lower RMSD values (average distance between the atoms of superimposed proteins), TcaB was greatly aligned with 4O9Y followed by 6OGD, 1J0M, and 1I5P (Supplementary Figure S5). On the contrary, TcaA protein of *P.akhurstii* (used as the control) showed greater RMSD values and lower % similarity when aligned with 1J0M, 1I5P, 6OGD and 4O9Y (Supplementary Figure S5). The greatest similarity of both TcaB and TcaA with 4O9Y is not surprising maybe because of the prevalence of Tc gene isoforms in the *Photorhabdus* genome [13].

Additionally, using protein-protein docking analysis, we assumed that TcaB putatively binds toward the C-terminal end of GmCAD protein via a number of salt bridge, hydrogen bond, and pi-alkyl interactions (Supplementary Figure S6).

### *TcaB was resistant to gut juice digestion* in vitro

**Figure 5.**
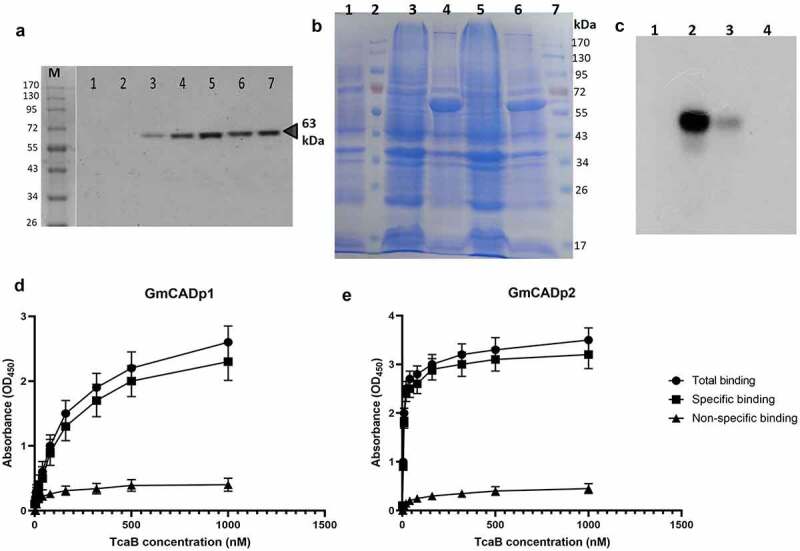
**Effect of insect gut juice on TcaB integrity and analysis of TcaB binding with GmCAD peptides via ligand blot and ELISA assay**. (a) Western blot analysis of purified TcaB protein treated with *G. mellonella* gut juice using the anti-TcaB antibody. M, protein marker. Lanes: 1,2 – gut juice; 3 – purified TcaB; 4 – gut juice: purified TcaB (1: 1); 5 – gut juice: purified TcaB (2: 1); 6 – gut juice: purified TcaB (4: 1); 7 – gut juice: purified TcaB (8: 1). (b) SDS-PAGE-based detection of overexpressed GmCAD fragments. Lanes: 1, total proteins in inclusion bodies of untransformed *E. coli*; 2 and 7, protein markers; 3 and 4, total proteins in inclusion bodies of *E. coli* transformed with pET-GmCADp2 before and after IPTG induction (62 kDa); 5 and 6, total proteins in inclusion bodies of *E. coli* transformed with pET-GmCADp1 before and after IPTG induction (60 kDa). (c) Ligand blot analysis of TcaB binding with GmCAD fragments. Lanes: 1, protein marker; 2, GmCADp2; 3, GmCADp1; 4, bovine serum albumin (BSA) as a control. Binding ELISA was carried out by fixing 1 µg of GmCADp1. (d) GmCADp2. (e) Fragments in microtiter plate wells followed by incubation with increasing concentrations of biotinylated TcaB protein. *K*_d_ values obtained using GraphPad Prism 9.0.0 analysis are indicated within the graphs

Purified TcaB toxin was digested with *G. mellonella* midgut juice at 37°C in alkaline pH using different gut juice/toxin ratios. Western blot analysis showed that TcaB was resistant to further degradation when suspended in the gut juice (Figure 5a), which constitutes a number of proteases, suggesting that the putatively protease resistant TcaB core needs no further processing when delivered into the insect gut. As control, a purified Cry1Ac protoxin (kindly provided by Dr. Rohini Sreevathsa, National Institute for Plant Biotechnology, New Delhi; details of the toxin *in vitro* production are provided in the Indian patent number 237912) was treated with *G. mellonella* gut juice in different ratios as described above. SDS-PAGE analysis showed that 130 kDa protoxin was cleaved into a number of protein fragments ranging from 65–100 kDa upon digestion with the gut juice (Supplementary Figure S7).

### TcaB protein binds to recombinant GmCAD peptides

Two cDNA fragments of *GmCAD* gene, *GmCADp1* (1662 bp) and *GmCADp2* (1695 bp), were cloned and expressed in *E. coli* BL21 (DE3) cells. These cDNA fragments conceptually translate into peptides of 554 and 565 amino acid residues with molecular masses of 60 and 62 kDa, respectively. Protein overexpression in the pelleted inclusion bodies was confirmed by SDS-PAGE (Figure 5b). The measured molecular mass of the peptides was within the instrumental error (0.05%) of predicted molecular mass. LC-MS/MS (Q-TOF)-based mass-spectroscopy of the in-gel tryptic digests indicated that these peptides are parts of the GmCAD protein. Specific interaction between TcaB protein and GmCAD was investigated by ligand blot assay. Although both the expressed GmCAD fragments bound to the activated TcaB, the binding affinity of GmCADp2 was comparatively greater than that of GmCADp1 as higher hybridization (banding) intensity of the former was detected in the blot (Figure 5c).

Subsequently, binding potential of GmCAD fragments to TcaB was examined by ELISA. According to the calculated dissociation constant (*K*_d_) values, TcaB bound to GmCADp1 with lower affinity (*K*_d_ for specific binding =_ _8.09 ± 3.43 nM, *R^2^* = 0.981) compared to GmCADp2 (*K*_d_ for specific binding =_ _189.50 ± 55.69 nM, *R^2^* = 0.998) (Figure 5d,e).

### *RNAi knockdown of* GmCAD *transcript*

**Figure 6.**
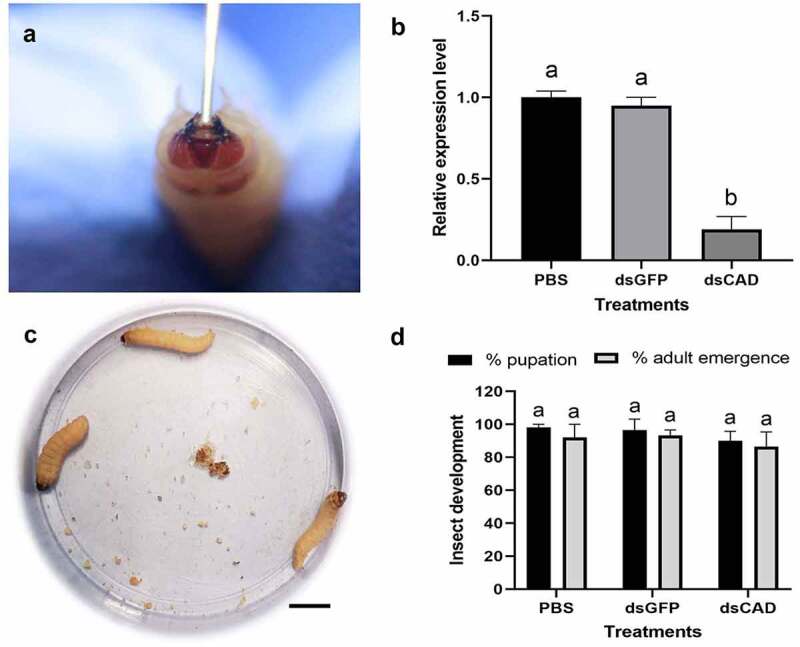
**RNAi knockdown of *GmCAD* transcript in the fourth-instar larvae of *G. mellonella***. (a) Oral administration of *GmCAD* dsRNA-expressing HT115 clone using a hypodermic syringe. (b) RT-qPCR-based detection of *GmCAD* transcript in *GmCAD* or *gfp* dsRNA-treated larval midgut at 24 h after inoculation. Larvae force fed with PBS were used as the negative control. Gene expression was normalized using *G. mellonella 18S rRNA* and *EF-1α* genes. Each bar represents the relative expression value with standard error of RT-qPCR runs in three biological (consisting of 15–20 larvae) and three technical replicates. Different letters indicate significant differential expression at p < 0.01, Tukey’s HSD test. (c) No morphological/behavioral aberration was observed in *GmCAD* dsRNA-treated larvae feeding on artificial diet at 2 days after inoculation. Scale bar = 1 cm. (d) Per cent pupation and adult emergence data of dsRNA-treated insects at 7–10 days after inoculation. Each bar with same letter is indicative of no significant difference (p > 0.01, Tukey’s HSD test) between treatments (*n* = 20; three biological replicates)

Bacterially expressed CAD dsRNAs (~ 10 µg/20 µL PBS) were orally administered into *G. mellonella* fourth-instar larvae using a hypodermic syringe (Figure 6a). Post inoculation larvae were reared on an artificial diet. At 24 h after inoculation, *GmCAD* transcripts in larval gut were significantly (*F*_2,18_ = 24.44, p < 0.0001) reduced by approximately 80%, compared to that in the larvae force fed with PBS or *gfp* dsRNA (Figure 6b). The suppression of *GmCAD* expression did not affect insect behavior and development in terms of their pupation and adult emergence ratio (Figure 6c,d).

### *RNAi of* GmCAD *reduced* G. mellonella *susceptibility to TcaB*

**Figure 7.**
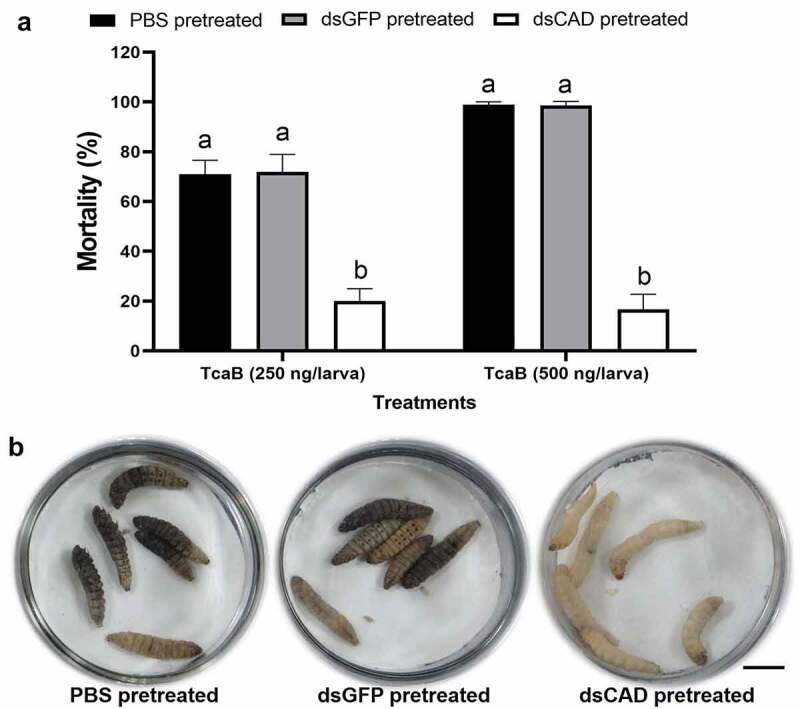
**Silencing of *GmCAD* expression alters *G. mellonella* susceptibility to TcaB toxin**. (a) Per cent mortality of TcaB-treated larvae pre-treated with *GmCAD* or *gfp* dsRNA. After 24 h of dsRNA treatment, lethal (250 ng/larva) and sub-lethal (500 ng/larva) dose of TcaB was orally administered as described in Figure 6. After another 24 h mortality data was recorded. Larvae force fed with PBS were used as the negative control. Bars with different letters denote significant difference (p < 0.01, Tukey’s HSD test) between treatments (*n* = 30; three biological replicates). (b) PBS or *gfp* dsRNA pre-treated larvae when force fed with lethal dose of TcaB exhibited dead or morbid phenotypes (indicative of dark cuticular melanization, were unresponsive to touch), in contrast to normal phenotypes in *GmCAD* dsRNA pre-treated larvae force fed with lethal dose of TcaB

Larvae pretreated with CAD dsRNA for 24 h were force fed with TcaB toxin in lethal and sub-lethal concentrations, and larval mortality data was recorded after another 24 h. Compared to PBS and *gfp* dsRNA pretreated larvae, mortality in CAD dsRNA pretreated larvae reduced by approximately 72 (*F*_2,27_ = 54.78, p < 0.0001) and 83% (*F*_2,27_ = 59.22, p < 0.0001) when ingested with 250 and 500 ng/larva TcaB dose, respectively (Figure 7a). The qualitative demonstration of toxin susceptibility reduction in RNAi insects is provided in Figure 7b.

## Discussion

The midgut CAD receptors located in the apical surface of epithelial cells are known to interact with Cry toxins to induce Bt intoxication in a number of lepidopteran insects [27–29]. Nevertheless, no information is available on molecular characteristics of CAD protein in the model insect, *G. mellonella*. Current study reports the complete ORF of GmCAD, which consists of extracellular (1–1690 aa), transmembrane (1691–1710 aa), and intracellular (1711–1840 aa) domains. Fourteen cadherin repeats (CRs) were predicted in the extracellular domain. Interestingly, among lepidopteran CADs, a number of CRs (*B. mori* – 9, *O. nubilalis, S. exigua, Pectinophora gossypiella, Heliothis virescens* – 11, *Helicoverpa armigera, M. sexta* – 12, *C. suppressalis* – 14) vary considerably [29,30], indicating its species-specific conservation. Fifty-four CAD sequences from different insect species were used to construct a maximum likelihood method-based phylogenetic tree, which showed that lepidopteran CAD sequences are highly conserved as they form a distinct clade that diverges from hymenopteran, isopteran, coleopteran and hemipteran clades.

GmCAD contains a number of N-glycosylated asparagine residues at 71, 185, 221, 314, 419, 520, 665, 735, 809, 858, 957, 977, 1048, 1078, 1186, 1312, 1344, 1398, 1433, 1491 and 1521 aa positions, and a number of O-glycosylated serine/threonine residues at 335, 911, 1789 and 1824 aa positions. The presence of N-Acetylgalactosamine (GalNAc) residues in GmCAD is quite intriguing because it has been found recently that TcdA1 toxin from *P. luminescens* binds with N-glycan sugars with GalNAc residues in order to induce Tc toxin sensitivity in host cells [31].

**Figure 8.**
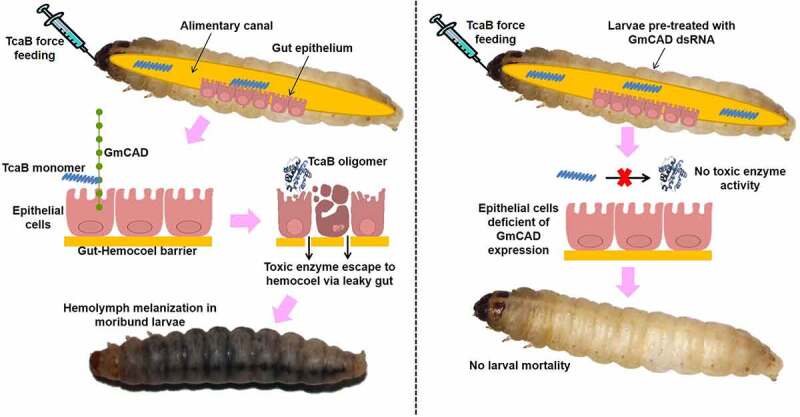
**Schematic representation of TcaB mode of action in control (left panel) and dsRNA-treated (right panel) *G. mellonella***. In control insects, upon oral ingestion the protease resistant core of TcaB monomer reaches midgut epithelial membrane and binds to transmembrane GmCAD receptor. This leads to TcaB oligomerization and toxic enzyme activity on epithelial cells. After degeneration of gut epithelium, TcaB escapes to hemocoel via leaky gut and induces extensive hemolymph melanization toward larval mortality. By contrast, in RNAi insects, monomeric TcaB cannot interact with downregulated GmCAD in the midgut epithelium and due to defunct oligomerization cannot confer toxic enzyme activity

In order to investigate the physiological roles of GmCAD in *G. mellonella*, the expression patterns of *GmCAD* mRNA was extensively analyzed. *GmCAD* was highly expressed in fourth-instar larval stage compared to other life stages. Furthermore, *GmCAD* was most abundantly expressed in the midgut tissues compared to other body parts such as Malpighian tubule, foregut, hindgut, head, and fat body. A similar expression patterns of CAD was detected in other lepidopteran insects [30,32–35]. Incidentally, we found that the *G. mellonella* fourth-instar stage was most vulnerable to *P. akhurstii* TcaB intoxication and TcaB catalytic activity in the midgut epithelium [21,22]. Thus, we hypothesized that the activated TcaB monomer may bind to GmCAD, which facilitates TcaB oligomerization leading to toxic enzyme activity (Figure 8). Herein, we demonstrate that the truncated TcaB (63 kDa) toxin we used was resistant to protease digestion when suspended in *G. mellonella* gut juice (in alkaline pH), suggesting that it was delivered in *G. mellonella* in its activated form. Notably, a 70 kDa Cry2Ab short protoxin is activated by midgut proteases to provide a 65 kDa protease-resistant core after removal of 40 amino acids from the N-terminal end [36].

In order to validate our hypothesis that GmCAD acts as a functional receptor of TcaB toxin, we performed ligand binding and ELISA assay. In line with the revelation that C-terminal CRs are the favorable interacting sites for Cry proteins [29], we synthesized two GmCAD peptides (GmCADp1: CR6-CR10, distal from transmembrane domain and GmCADp2: CR11-MPED, proximal to transmembrane domain) for comparative binding analysis. Our ligand blot and binding ELISA assays indicated that GmCADp2 binds with TcaB with higher affinity compared to lower binding affinity between GmCADp1 and TcaB. Our *in silico* study indicated that TcaB docked with GmCAD toward the C-terminal end via a number of salt-bridge, hydrogen bond and pi-alkyl interactions.

As a second line of evidence to establish the functional role of GmCAD in TcaB intoxication, we performed RNAi knockdown of *GmCAD* gene in *G. mellonella*. Oral feeding of bacterially expressed dsRNA was highly effective to suppress *GmCAD* mRNA levels in the midgut tissue. Subsequently, the susceptibility of dsRNA-treated insects to TcaB toxin was markedly reduced as revealed by the mortality data. Similar RNAi silencing of CAD transcription in a number of lepidopteran insects also led to reduced sensitivity to Cry toxins, implying that CAD is a functional receptor of gut-active toxins [29,30,32–35]. Expression interference of *GmCAD* did not cause any unintended off-target effect as *GmCAD* suppression did not affect pupal and adult development of *G. mellonella* in our study. Since CADs are involved in calcium-dependent cell-cell adhesion, no adverse effect on subsequent metamorphosis is quite intriguing. We assume that *GmCAD* RNAi had no effect during midgut tissue regeneration in subsequent metamorphic development of *G. mellonella*. Notably, RNAi efficiency varies with species, targeted tissue, and target genes in lepidopteran insects [37,38]. *GmCAD* share 97.94% identity with another *G. mellonella* CAD (XP_026759573.1) and only five nucleotides are different in the region where dsRNA was designed, implicating that RNAi of *GmCAD* might have silenced its other allelic variant. However, we could not prove this experimentally due to our inability to design primers that would discriminate between the two highly homologous transcripts.

In conclusion, our results demonstrate the involvement of GmCAD as one of the functional receptor in *P. akhurstii* TcaB-induced toxicity in *G. mellonella*. During the intoxication process, the gut-active TcaB monomer binds to the transmembrane-proximal region of GmCAD, which putatively leads to TcaB oligomerization and induce its catalytic activity on the epithelial cells toward gut leakiness (Figure 8). Using domain conservation analysis and secondary structure alignment, we showed that *P. akhurstii* TcaB has high degree of similarity to *Bacillus* sp. crystal protein, *B. thuringiensis* Cry2Aa toxin, *Y. entomophaga* Tc toxin, and *P. luminescens* TcdA toxin. Although the complete pathway is not yet elucidated, we assume that *P. akhurstii* TcaB mimics the Cry intoxication mechanism in *G. mellonella*. Present finding forms the basis of future research related to the insect gut receptor interactions with *Photorhabdus* toxins.

## Supplementary Material

Supplemental Material

## Data Availability

Datasets are available on request. The raw data related to this manuscript will be made available by the corresponding author to any qualified researcher upon request.

## References

[1] Garcia-del-pino F, Morton A, Shapiro-Ilan D. Entomopathogenic nematodes as biological control agents of tomato pests. In: Wakil W, Brust GE, Perring TM, editors. Sustainable management of arthropod pests of tomato. Academic Press, Elsevier; 2018. p. 269–282. https://www.sciencedirect.com/science/article/pii/B9780128024416000127

[2] Sajnaga E, Kazimierczak W. Evolution and taxonomy of nematode-associated entomopathogenic bacteria of the genera *Xenorhabdus* and *Photorhabdus*: an overview. Symbiosis. 2020;80(1):1–13.

[3] Clarke DJ. *Photorhabdus*: a tale of contrasting interactions. Microbiology. 2020;166:335–348. 10.1099/mic.0.000907 32209172

[4] Machado RA, Thönen L, Arce CC, et al. Engineering bacterial symbionts of nematodes improves their biocontrol potential to counter the western corn rootworm. Nat Biotechnol. 2020;38(5):600–608. 32066956 10.1038/s41587-020-0419-1

[5] Ahuja A, Kushwah J, Mathur C, et al. Identification of Galtox, a new protein toxin from *Photorhabdus* bacterial symbionts of *Heterorhabditis* nematodes. Toxicon. 2021;194:53–62. 33610634 10.1016/j.toxicon.2021.02.011

[6] Dutta TK, Mathur C, Mandal A, et al. The differential strain virulence of the candidate toxins of *Photorhabdus akhurstii* can be correlated with their inter-strain gene sequence diversity. 3 Biotech. 2020;10:299. 10.1007/s13205-020-02288-0 PMC7286996 32550116

[7] Dutta TK, Santhoshkumar K, Mathur C, et al. A Photorhabdus akhurstii toxin altered gut homeostasis prior conferring cytotoxicity in *Spodoptera frugiperda, S*. litura and *Helicoverpa armigera*. Phytoparasitica. 2021;49:943–958.

[8] Ffrench-constant RH, Dowling A, Waterfield NR. Insecticidal toxins from *Photorhabdus* bacteria and their potential use in agriculture. Toxicon. 2007;49(4):436–451. 17207509 10.1016/j.toxicon.2006.11.019

[9] Mathur C, Kushwah J, Somvanshi VS, et al. A 37 kDa Txp40 protein characterized from *Photorhabdus luminescens* sub sp. *akhurstii* conferred injectable and oral toxicity to greater wax moth, *Galleria mellonella*. Toxicon. 2018;154:69–73. 30278182 10.1016/j.toxicon.2018.09.007

[10] Waterfield NR, Ciche T, Clarke D. *Photorhabdus* and a host of hosts. Annu Rev Microbiol. 2009;63(1):557–574. 19575559 10.1146/annurev.micro.091208.073507

[11] Roderer D, Hofnagel O, Benz R, et al. Structure of a Tc holotoxin pore provides insights into the translocation mechanism. Proc Natl Acad Sci USA. 2019;116(46):23083–23090. 31666324 10.1073/pnas.1909821116 PMC6859359

[12] Roderer D, Raunser S. Tc toxin complexes: assembly, membrane permeation, and protein translocation. Ann Revof Microbiol. 2019;73(1):247–265. 10.1146/annurev-micro-102215-095531 31140906

[13] Sheet J, Aktories K. Insecticidal toxin complexes from *Photorhabdus luminescens*. In: Ffrench-constant RH, editor. The molecular biology of *Photorhabdus* bacteria. Cham: Springer; 2017. p. 3–24. 10.1007/82_2016_55 28233068

[14] Jang E-K, Jung BK, Park G-S, et al. Cloning and expression of the insecticidal toxin gene “tccB” from *Photorhabdus temperata* M1021 in *Escherichia coli* expression system. J Asia-Pac Entomol. 2020;23(1):172–176.

[15] Blackburn MB, Martin PA, Kuhar D, et al. The occurrence of Photorhabdus-like toxin complexes in Bacillus thuringiensis. PLoS One. 2011;6:e18122. 21464948 10.1371/journal.pone.0018122 PMC3064592

[16] Morgan JA, Sergeant M, Ellis D, et al. Sequence analysis of insecticidal genes from *Xenorhabdus nematophilus* PMFI296. Appl Environ Microbiol. 2001;67(5):2062–2069. 11319082 10.1128/AEM.67.5.2062-2069.2001 PMC92837

[17] Waterfield NR, Hares M, Yang G, et al. Potentiation and cellular phenotypes of the insecticidal toxin complexes of *Photorhabdus* bacteria. Cell Microbiol. 2005;7(3):373–382. 15679840 10.1111/j.1462-5822.2004.00467.x

[18] Yang G, Waterfield NR, Ausubel FM. The role of TcdB and TccC subunits in secretion of the *Photorhabdus* Tcd toxin complex. PLoS Pathog. 2013;9(10):e1003644. 24098116 10.1371/journal.ppat.1003644 PMC3789776

[19] Liu D, Burton S, Glancy T, et al. Insect resistance conferred by 283-kDa *Photorhabdus luminescens* protein TcdA in *Arabidopsis thaliana*. Nat Biotechnol. 2003;21(10):1222–1228. 12949536 10.1038/nbt866

[20] Guo L, Fatig RO, Orr GL, et al. *Photorhabdus luminescens* W-14 insecticidal activity consists of at least two similar but distinct proteins. Purification and characterization of toxin A and toxin B. J Biol Chem. 1999;274(14):9836–9842. 10092674 10.1074/jbc.274.14.9836

[21] Mathur C, Phani V, Kushwah J, et al. TcaB, an insecticidal protein from *Photorhabdus akhurstii* causes cytotoxicity in the greater wax moth, *Galleria mellonella*. Pestic Biochem Physiol. 2019;157:219–229. 31153472 10.1016/j.pestbp.2019.03.019

[22] Santhoshkumar K, Mathur C, Mandal A, et al. A toxin complex protein from *Photorhabdus akhurstii* conferred oral insecticidal activity against *Galleria mellonella* by targeting the midgut epithelium. Microbiol Res. 2021;242:126642. 33191102 10.1016/j.micres.2020.126642

[23] Dutta TK, Lovegrove A, Gaur HS, et al. Differential immunoreactivity of the root-knot nematodes, *Meloidogyne graminicola* and *Meloidogyne incognita* to polyclonal and monoclonal antibodies and identification of antigens through proteomics approach. Afr J Microbiol Res. 2014;8:1245–1254.

[24] Shankhu PY, Mathur C, Mandal A, et al. Txp40, a protein from Photorhabdus akhurstii, conferred potent insecticidal activity against the larvae of Helicoverpa armigera, Spodoptera litura and S. exigua. Pest Manag Sci. 2020;76(6):2004–2014. 31867818 10.1002/ps.5732

[25] Dubovskiy IM, Grizanova EV, Whitten MM, et al. Immuno-physiological adaptations confer wax moth *Galleria mellonella* resistance to *Bacillus thuringiensis*. Virulence. 2016;7:860–870. 27029421 10.1080/21505594.2016.1164367 PMC5160394

[26] Grizanova EV, Coates CJ, Dubovskiy IM, et al. Metarhizium brunneum infection dynamics differ at the cuticle interface of susceptible and tolerant morphs of Galleria mellonella. Virulence. 2019;10(1):999–1012. 31724467 10.1080/21505594.2019.1693230 PMC8647853

[27] Adang MJ, Crickmore N, Jurat-Fuentes JL. Diversity of *Bacillus thuringiensis* crystal toxins and mechanism of action. In: Dhadialla TS, Gill SS, editors. Advances in insect physiology: insect midgut and insecticidal proteins. Vol. 47. San Francisco: Elsevier; 2014. p. 39–87.

[28] Bravo A, Likitvivatanavong S, Gill SS, et al. *Bacillus thuringiensis*: a story of a successful bioinsecticide. Insect Biochem Mol Biol. 2011;41:423–431. 21376122 10.1016/j.ibmb.2011.02.006 PMC3689885

[29] Fabrick JA, Wu Y. Roles of insect midgut cadherin in Bt intoxication and resistance. In: Soberón M, Gao Y, Bravo A, editors. Bt resistance: characterization and strategies for GM crops producing *Bacillus thuringiensis* toxins. Wallingford/ Boston: CABI; 2015. p. 69–86.

[30] Zhou H, Hu W, Huang Q, et al. Knockdown of cadherin genes decreases susceptibility of Chilo suppressalis larvae to Bacillus thuringiensis produced Crystal toxins. Insect Mol Biol. 2020;29(3):301–308. 31908051 10.1111/imb.12634

[31] Ng’ang’a PN, Siukstaite L, Lang AE, et al. Involvement of N-glycans in binding of *Photorhabdus luminescens* Tc toxin. Cell Microbiol. 2021;23(8):e13326. 33720490 10.1111/cmi.13326

[32] Park Y, Kim Y. RNA interference of cadherin gene expression in *Spodoptera exigua* reveals its significance as a specific Bt target. J Invertebr Pathol. 2013;114(3):285–291. 24055650 10.1016/j.jip.2013.09.006

[33] Qiu L, Hou L, Zhang B, et al. Cadherin is involved in the action of *Bacillus thuringiensis* toxins Cry1Ac and Cry2Aa in the beet armyworm, *Spodoptera exigua*. J Invertebr Pathol. 2015;127:47–53. 25754522 10.1016/j.jip.2015.02.009

[34] Ren XL, Chen RR, Zhang Y, et al. A *Spodoptera exigua* cadherin serves as a putative receptor for *Bacillus thuringiensis* Cry1Ca toxin and shows differential enhancement of Cry1Ca and Cry1Ac toxicity. Appl Environ Microbiol. 2013;79(18):5576–5583. 23835184 10.1128/AEM.01519-13 PMC3754171

[35] Zhao M, Yuan X, Wei J, et al. Functional roles of cadherin, aminopeptidase-N and alkaline phosphatase from *Helicoverpa armigera* (Hübner) in the action mechanism of *Bacillus thuringiensis* Cry2Aa. Sci Rep. 2017;7(1):46555. 28488696 10.1038/srep46555 PMC5424343

[36] Liu S, Wang S, Wu S, et al. Proteolysis activation of Cry1Ac and Cry2Ab protoxins by larval midgut juice proteases from Helicoverpa armigera. Plos One. 2020;15(1):e0228159. 32004347 10.1371/journal.pone.0228159 PMC6994024

[37] Chen L, Wei J, Liu C, et al. Specific binding protein ABCC1 is associated with Cry2Ab toxicity in *Helicoverpa armigera*. Front Physiol. 2018;9:745. 29971014 10.3389/fphys.2018.00745 PMC6018205

[38] Terenius O, Papanicolaou A, Garbutt JS, et al. RNA interference in Lepidoptera: an overview of successful and unsuccessful studies and implications for experimental design. J Insect Physiol. 2011;57(3):231–245. 21078327 10.1016/j.jinsphys.2010.11.006

