# Supplementary material for: The induced knockdown of GmCAD receptor protein encoding gene in Galleria mellonella decreased the insect susceptibility to a Photorhabdus akhurstii oral toxin
Source: Virulence. 2021 Dec 9;12(1):2957–71. doi: 10.1080/21505594.2021.2006996 (PMC8667893; doi:10.1080/21505594.2021.2006996)

**SUPPLEMENTARY FIGURES**

*Title:* **The induced knockdown of GmCAD receptor protein encoding gene in *Galleria mellonella* decreased the insect susceptibility to a *Photorhabdus akhurstii* oral toxin**

*Short title:* Receptor gene knockdown reduce insect susceptibility to bacterial toxin

*Authors:* Tushar K. Dutta^1^*, Arudhimath Veeresh^1^, Chetna Mathur^1^, Victor Phani^2^, Abhishek Mandal^3^, Doddachowdappa Sagar^4^, Suresh M. Nebapure^4^

*Affiliation:* ^1^Division of Nematology, ICAR-Indian Agricultural Research Institute, New Delhi, 110012, India

^2^Department of Agricultural Entomology, College of Agriculture, Uttar Banga Krishi Viswavidyalaya, Dakshin Dinajpur, West Bengal, India

^3^Division of Agricultural Chemicals, ICAR-Indian Agricultural Research Institute, New Delhi, 110012, India

^4^Division of Entomology, ICAR-Indian Agricultural Research Institute, New Delhi, 110012, India

*Corresponding Author*

^*^Dr. Tushar K Dutta

Division of Nematology

ICAR-Indian Agricultural Research Institute

New Delhi, India-110012

TEL: +91-11-2584-2721

Email: [tushar.dutta@icar.gov.in](mailto:tushar.dutta@icar.gov.in); [nemaiari@gmail.com](mailto:nemaiari@gmail.com)

**Supplementary Figure 1.** Total RNA extracted from the different body parts (of fourth-instar larvae) and developmental stages of *G. mellonella* was resolved onto a 2% (w/v) high resolution agarose gel. M – 1 Kb molecular weight marker. Lanes: 1, head; 2, fat body; 3, foregut; 4, midgut; 5, hindgut; 6, Malpighian tubule; 7, first-instar; 8, second-instar; 9, third-instar; 10, fourth-instar; 11, fifth-instar.


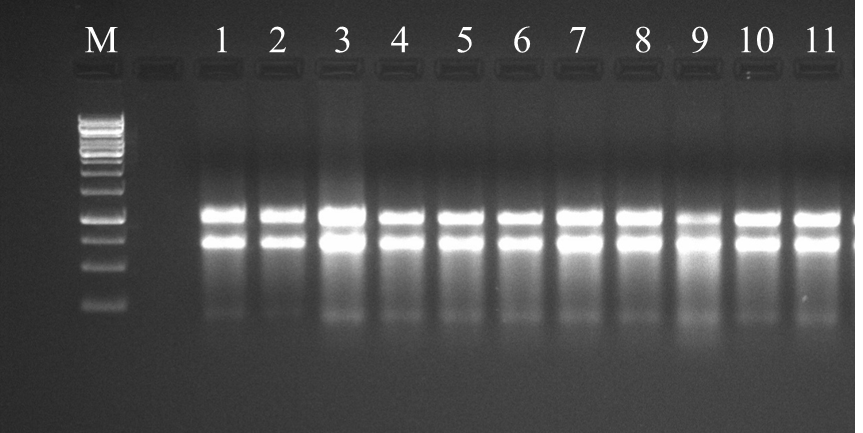


**Supplementary Figure 2. Confirmation of bacterially expressed dsRNA in agarose gel.** Recombinant HT115 cells were centrifuged at 5,000 g for 10 min. Cell pellets were resuspended in 1 M ammonium acetate/10 mM EDTA plus the identical volume of phenol:chloroform:isoamyl alcohol (25:24:1). The samples were incubated at 65° C for 30 min and centrifuged at 12,000 g for 15 min. The upper phase was mixed with isopropanol, incubated at -20 °C overnight and centrifuged at 12,000 g for 30 min. Obtained dsRNA was treated with RNase free DNase (Promega) to remove single strand DNA and RNase A solution (Promega) to remove single strand RNA. DsRNA pellets were resuspended in 1X TE buffer, pH 7.5 and loaded onto a 2% agarose TBE gel, stained with ethidium bromide, and photographed. DsRNA concentration was determined by Nanodrop 1000 (Thermo Fisher Scientific). M, 100 bp marker; Lanes 1-4, DsRNA purified from four independent cultures of IPTG-induced *E. coli* HT115 cells.


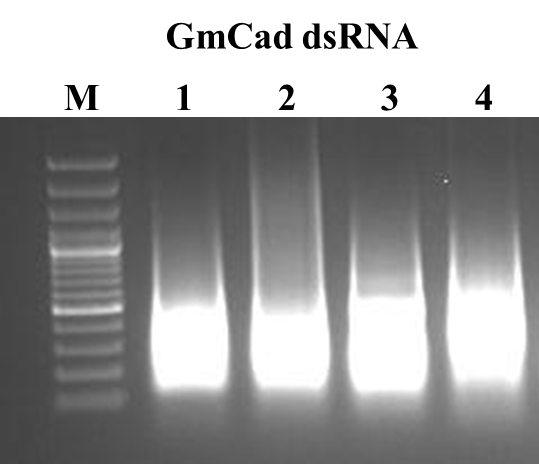


**Supplementary Figure 3.** Simulated binding site interactions of *P. akhurstii* TcaB and *G. mellonella* midgut proteins – α-amylase and prohibitin. 19 hydrogen bonds and 11 pi-alkyl bonds were detected in TcaB-α-amylase interface. 23 hydrogen bonds, 3 salt bridges and 11 pi-alkyl bonds were detected in TcaB-prohibitin interface. Protein-protein docking was performed in PatchDock webserver (<https://bioinfo3d.cs.tau.ac.il/PatchDock/>) and visualized in Discovery studio (v. 4.2). The best docking model was selected based on the geometric shape complementary score and the minimum energy potential among interacting molecules.

**
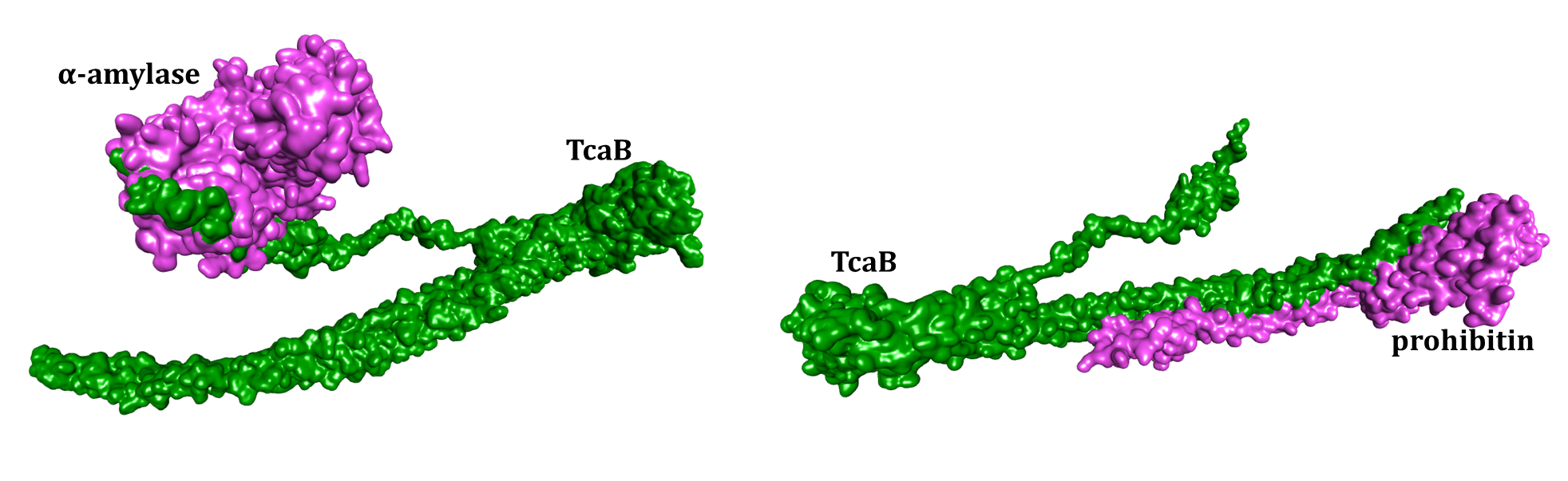
**

| **Receptor** | **Total Pi Interactions** | **Total Hydrogen Bonds** | **Total Salt Bridges** | **Ligand Contact Surface Area** | **Ligand Polar Contact Surface Area** | **Ligand Nonpolar Contact Surface Area** | **Receptor Contact Surface Area** | **Receptor Polar Contact Surface Area** | **Receptor Nonpolar Contact Surface Area** |
| --- | --- | --- | --- | --- | --- | --- | --- | --- | --- |
| α-amylase | 11 | 19 | 0 | 1623.4 | 647.17 | 976.19 | 1224.1 | 546.03 | 678.02 |

| **Receptor** | **Total Pi Interactions** | **Total Hydrogen Bonds** | **Total Salt Bridges** | **Ligand Contact Surface Area** | **Ligand Polar Contact Surface Area** | **Ligand Nonpolar Contact Surface Area** | **Receptor Contact Surface Area** | **Receptor Polar Contact Surface Area** | **Receptor Nonpolar Contact Surface Area** |
| --- | --- | --- | --- | --- | --- | --- | --- | --- | --- |
| Phohibitin | 2 | 23 | 3 | 1239.9 | 571.11 | 668.76 | 1260.3 | 584.77 | 675.56 |

**Supplementary Figure 4.** Amino acid sequence and domain structure of GmCAD from *Galleria mellonella*. The putative N-terminal signal peptide (SP, in brown, bold letter), 14 cadherin repeats (CR1-14), the membrane-proximal extracellular domain (MPED, in green, bold), the transmembrane domain (TM, in red, bold and italicized), and the internal cytoplasmic domain (IC, in purple, bold) are shown. Putative calcium-binding sites (DXNDN/DXD/LDRE/QAV/HAV) are underlined. Yellow highlighted regions indicate N- and O-Glycosylated residues.


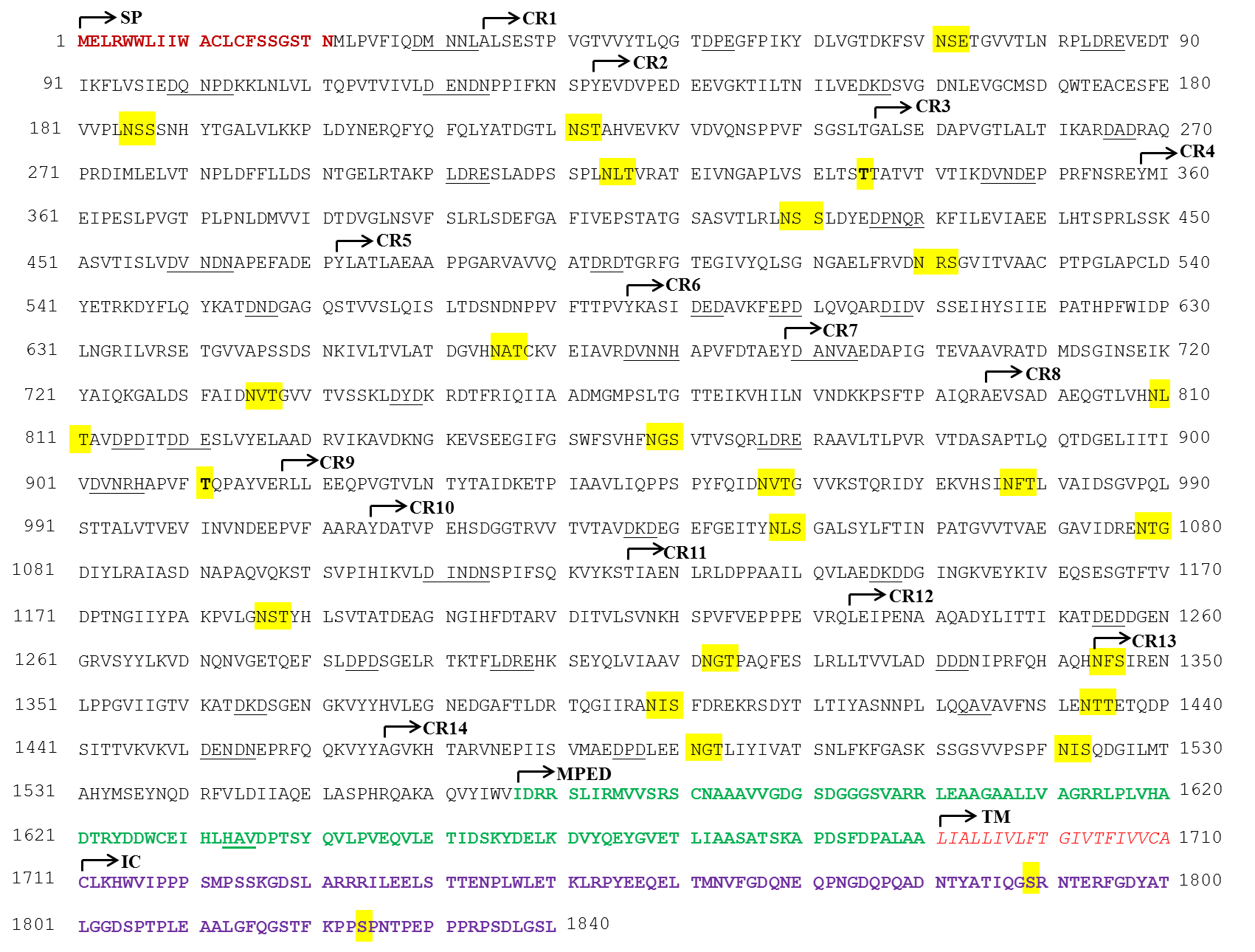


**Supplementary Figure 5.** Structure alignments of *P. akhurstii* TcaB with *Bacillus* sp. crystal protein (PDB accession number: 1J0M), *Bacillus thuringiensis* Cry2Aa toxin (1I5P), *Yersinia entomophaga* Tc toxin (6OGD) and *P. luminescens* TcdA toxin (4O9Y). *P. akhurstii* TcaA protein was used as the control. The 3D structures of TcaB and TcaA are represented as solid ribbon (red and sky blue colored ribbons indicate α-helix and β-sheet, respectively) and bond line in the top panel.. The 3D structure of 1J0M, 1I5P, 6OGD and 4O9Y and their comparative superimposition with TcaB and TcaA are shown in bottom panel. Superimposed protein structures were created in Discovery Studio using the jFAT-CAT algorithm. While superimposing, TcaB/TcaA structure is visualized as bond line and 1J0M, 1I5P, 6OGD and 4O9Y accessions are visualized as solid ribbon for clarity in viewing.


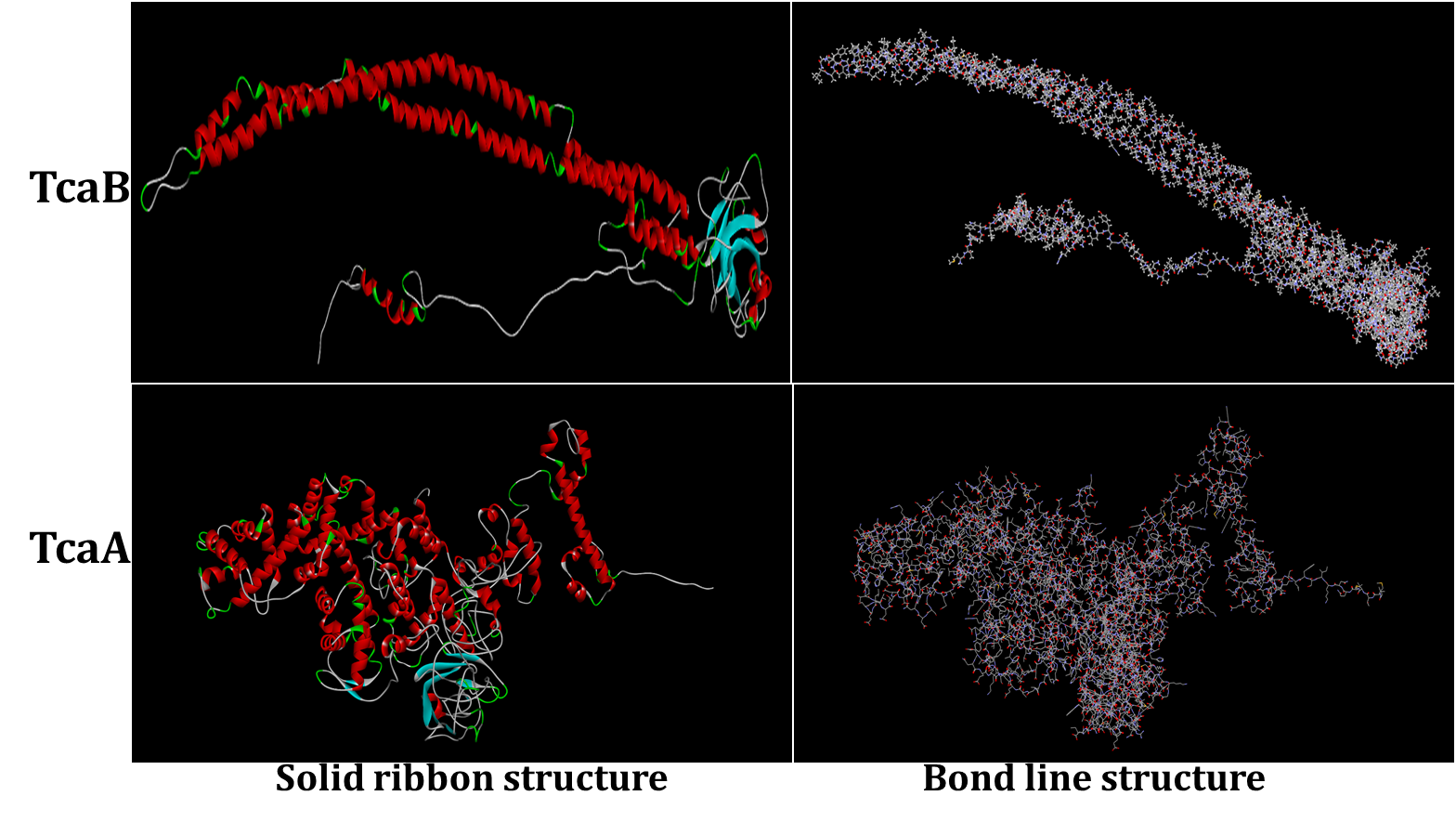


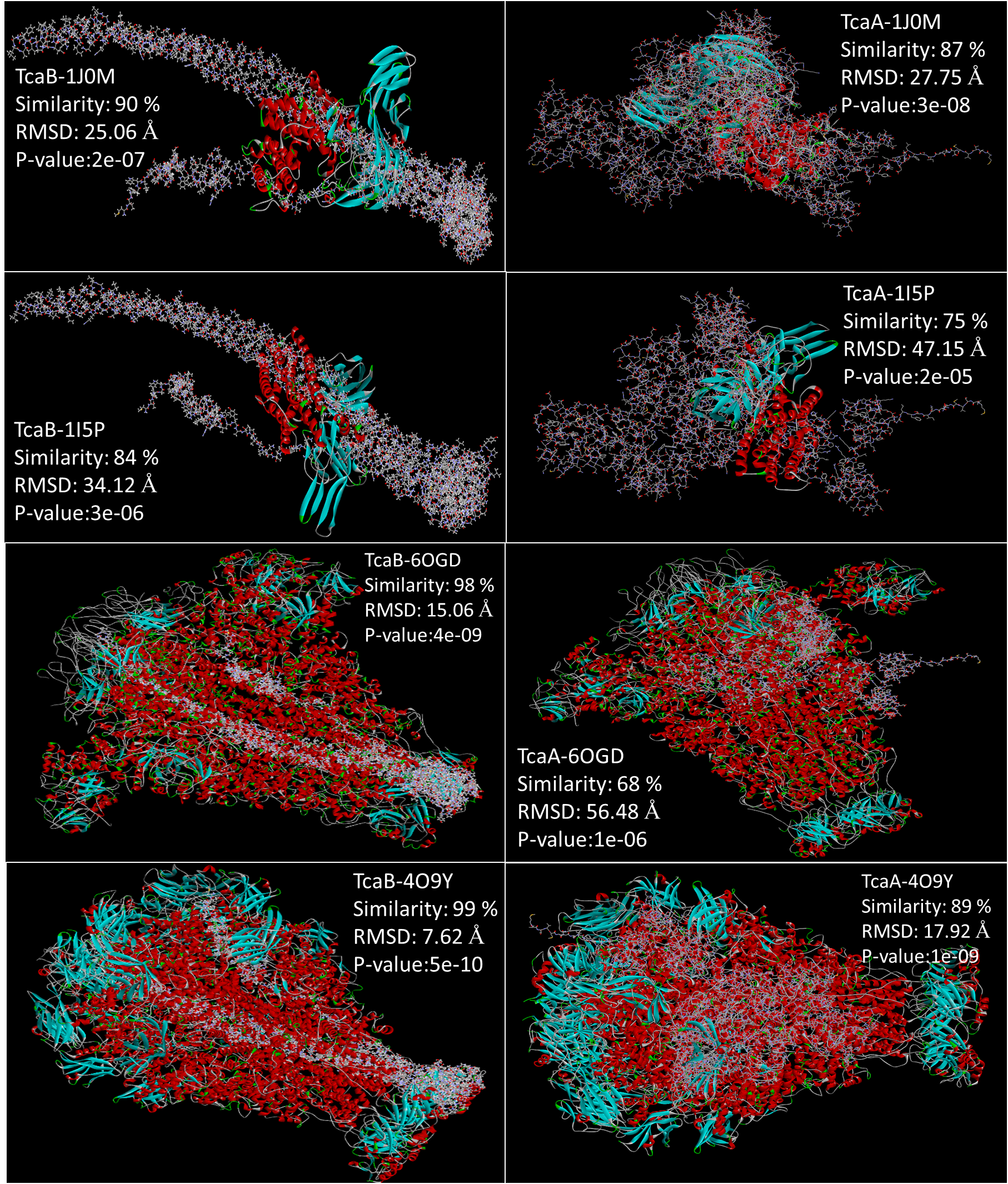


**Supplementary Figure 6.** The simulated binding site interactions between TcaB and GmCAD secondary structure. Red and sky blue colored ribbon indicate α-helix and β-sheet, respectively. **(A)** TcaB (predominantly alpha-helical) putatively binds towards the C-terminal end of GmCAD (predominantly beta-pleated). **(B)** The binding interface between two proteins is highlighted in yellow color. **(C)** Hydrophobic surface representation of the active sites present in TcaB molecule for probable binding to GmCAD. A number of salt bridge, hydrogen bond and pi-alkyl interactions were detected between TcaB-GmCAD complex.


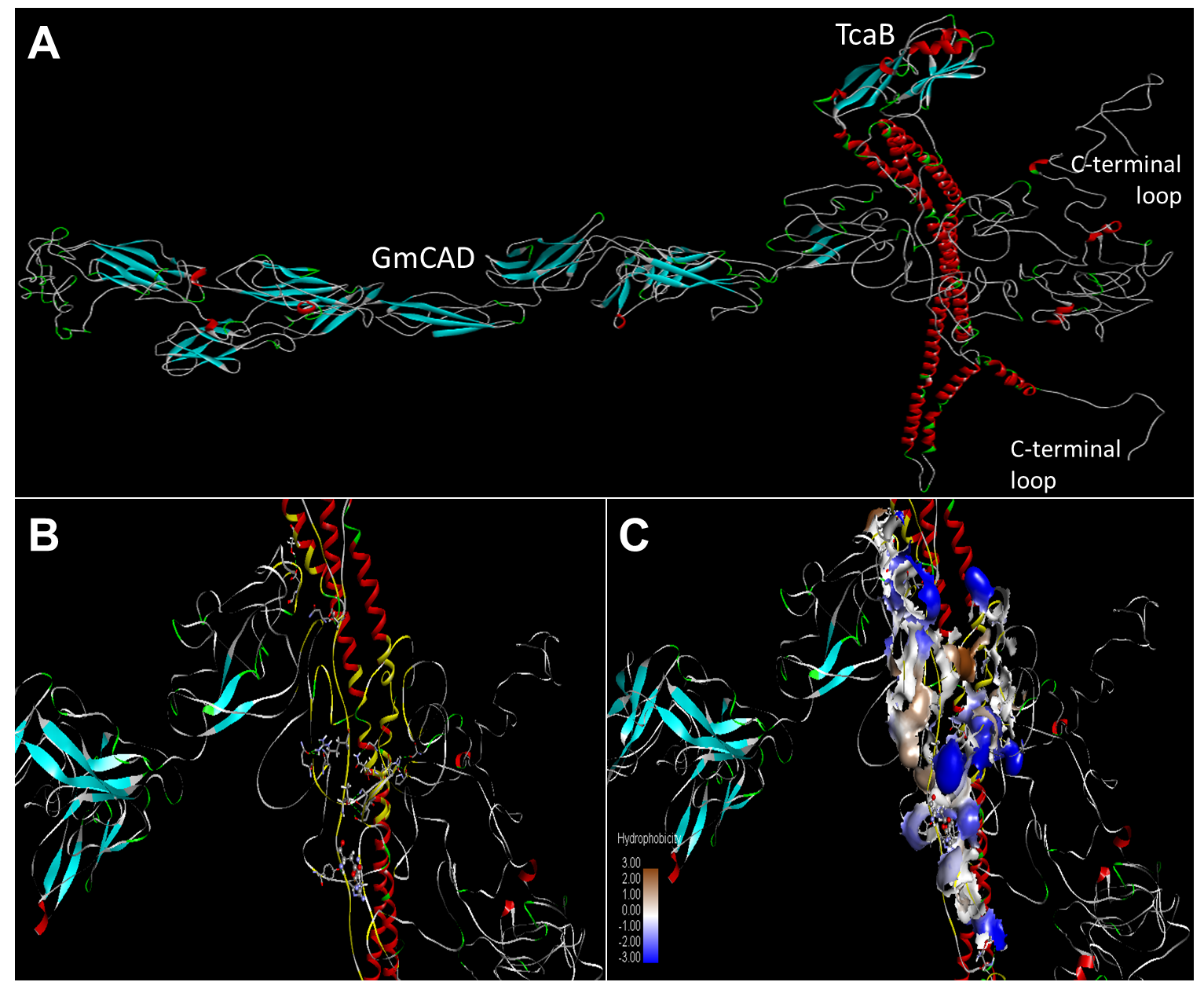


**Supplementary Figure 7.** Effect of insect gut juice on Cry1Ac protoxin. SDS-PAGE analysis shows that the 130 kDa protoxin was cleaved into a number of protein fragments ranging from 65-100 kDa upon digestion with *G. mellonella* gut juice. Lanes: 1 – purified Cry1Ac protoxin; 2 – gut juice: purified Cry1Ac (1: 1); 3 – gut juice: purified Cry1Ac (2: 1).


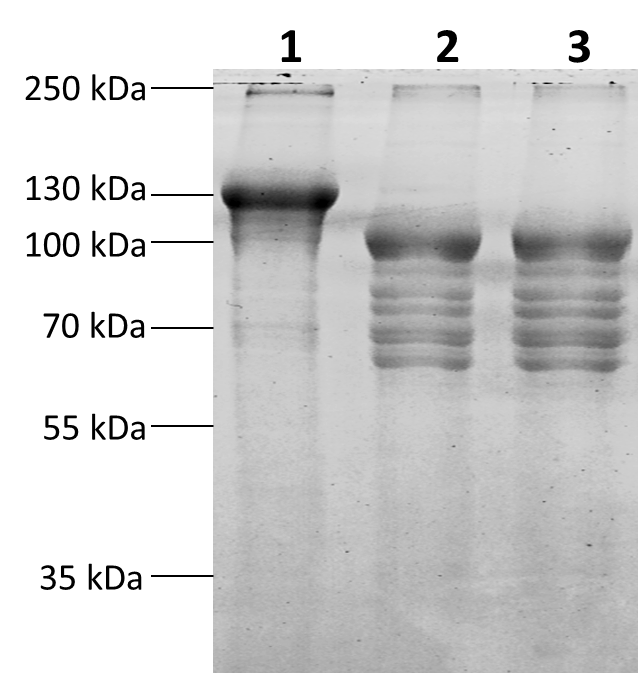

Supplement: Supplemental Material [file KVIR_A_2006996_SM0821.zip › supplementary/Supplementary_Figures_revised.docx]
