# Supplementary material for: The induced knockdown of GmCAD receptor protein encoding gene in Galleria mellonella decreased the insect susceptibility to a Photorhabdus akhurstii oral toxin
Source: Virulence. 2021 Dec 9;12(1):2957–71. doi: 10.1080/21505594.2021.2006996 (PMC8667893; doi:10.1080/21505594.2021.2006996)
Supplement: Supplemental Material [file KVIR_A_2006996_SM0821.zip › supplementary/Supplementary_Tables.docx]

**SUPPLEMENTARY TABLES**

*Title:* **The induced knockdown of GmCAD receptor protein encoding gene in *Galleria mellonella* decreased the insect susceptibility to a *Photorhabdus akhurstii* oral toxin**

*Short title:* Receptor gene knockdown reduce insect susceptibility to bacterial toxin

*Authors:* Tushar K. Dutta^1^*, Arudhimath Veeresh^1^, Chetna Mathur^1^, Victor Phani^2^, Abhishek Mandal^3^, Doddachowdappa Sagar^4^, Suresh M. Nebapure^4^

*Affiliation:* ^1^Division of Nematology, ICAR-Indian Agricultural Research Institute, New Delhi, 110012, India

^2^Department of Agricultural Entomology, College of Agriculture, Uttar Banga Krishi Viswavidyalaya, Dakshin Dinajpur, West Bengal, India

^3^Division of Agricultural Chemicals, ICAR-Indian Agricultural Research Institute, New Delhi, 110012, India

^4^Division of Entomology, ICAR-Indian Agricultural Research Institute, New Delhi, 110012, India

*Corresponding Author*

^*^Dr. Tushar K Dutta

Division of Nematology

ICAR-Indian Agricultural Research Institute

New Delhi, India-110012

TEL: +91-11-2584-2721

Email: [tushar.dutta@icar.gov.in](mailto:tushar.dutta@icar.gov.in); [nemaiari@gmail.com](mailto:nemaiari@gmail.com)

**Supplementary Table 1.** Oligonucleotides used for RACE, primer walking and verifying full-length cDNA sequence. T_m_ = 60°C

| **Primer (location in ORF)** | **Orientation** | **Sequence (5'-3')** | **Purpose** |
| --- | --- | --- | --- |
| GmCAD cDNA | Sense | GGATGCGAAAATAGGCGTAA | for obtaining first strand cDNA |
|  | Antisense | CGTTTATGCGATATTAGACCGTA |  |
| GmCAD GSP1* | Antisense | TGATTGGACGACGAGTTCAG | 5'-RACE |
| GmCAD NGSP1** | Antisense | TGGTTTTCCCAACTTCTTCGTC |  |
| GmCAD GSP2* | Sense | GGGGAGAATGGCAAAGTGTAC | 3'-RACE |
| GmCAD NGSP2** | Sense | ACACCACAGAAACCCAGGAC |  |
| Fragment 1 (5' UTR-315 aa) | Sense | GGATGCGAAAATAGGCGTAA | Primer walking for sequencing |
|  | Antisense | AAGGTTCAGTGGTGATGAAGG |  |
| Fragment 2 (305-636 aa) | Sense | AGCTGACCCTTCATCACCAC |  |
|  | Antisense | CTAAAATCCTGCCGTTGAGC |  |
| Fragment 3 (617-948 aa) | Sense | GATCATCGAACCTGCGACTC |  |
|  | Antisense | GCGGCTGGATGAGTACGG |  |
| Fragment 4 (925-1256 aa) | Sense | TGGGTACTGTCCTCAACACG |  |
|  | Antisense | TCCTCATCCGTCGCTTTTAT |  |
| Fragment 5 (1236-1568 aa) | Sense | CTGAGAATGCAGCGCAAG |  |
|  | Antisense | CGGTCGATCACCCATATGTA |  |
| Fragment 6 (1542 aa-3' UTR) | Sense | TCGTGTTGGACATCATAGCC |  |
|  | Antisense | CGTTTATGCGATATTAGACCGTA |  |
| GmCAD cDNA | Sense | CAGTGCTGTGATGTCGCTAGT | Full-length sequence verification |
|  | Antisense | AGGGTGGCGTGTCTTACATC |  |

*GSP, gene specific primer; **NGSP, nested gene specific primer.

**Supplementary Table 2.** Oligonucleotides used for peptide synthesis, RNAi and gene expression analysis. T_m_ = 60°C

| **Primer/gene/organism details** | **Orientation** | **Sequence (5'-3')** | **Purpose** | **PCR efficiency (%)** | **Standard curve *R*^2^** |
| --- | --- | --- | --- | --- | --- |
| GmCADp1 | Sense | GGATCCGACCGACTCCAACGACAACC ^a^ | Peptide expression | NA | NA |
|  | Antisense | AAGCTTAACGGAGGTTCTCCGCTATT ^b^ |  |  |  |
| GmCADp2 | Sense | GGATCCGCACAATAGCGGAGAACCTC ^a^ | Peptide expression | NA | NA |
|  | Antisense | AAGCTTGTGACGATGCCAGTGAACAG ^b^ |  |  |  |
| *GmCAD* | Sense | GAGCTCACCTACCACCTGTCGGTGAC ^c^ | dsRNA synthesis | NA | NA |
|  | Antisense | AAGCTTCAGCCAGCACCACTGATAGA ^b^ |  |  |  |
| *gfp* | Sense | GAGCTCGCAGAGCGAGGTATGTAGGC ^c^ | dsRNA synthesis | NA | NA |
|  | Antisense | AAGCTTCTGCCTCGGTGAGTTTTCTC ^b^ |  |  |  |
| *GmCAD* | Sense | ACCTGAAGGTGGACAACCAG | RT-qPCR | 105.7 | 0.992 |
|  | Antisense | CAGCCAGCACCACTGATAGA |  |  |  |
| *18S rRNA* (*G. mellonella*) | Sense | CACATCCAAGGAAGGCAG | RT-qPCR | 107.9 | 0.978 |
|  | Antisense | AGTGTACTCATTCCGATTACGA |  |  |  |
| *EF-1α* (*G. mellonella*) | Sense | AACCTCCTTACAGTGAATCC | RT-qPCR | 106.4 | 0.969 |
|  | Antisense | ATGTTATCTCCGTGCCAG |  |  |  |
| ABCC (*G. mellonella*) | Sense | CGTAACGATGATGTGCCAAC | RT-qPCR | 105.2 | 0.911 |
|  | Antisense | TGGAGGAACATGAATGCGTA |  |  |  |
| APN (*G. mellonella*) | Sense | GTCCTTCATCCGTGAGTGGT | RT-qPCR | 102.6 | 0.945 |
|  | Antisense | GGCAGCAACTTCGTCTAAGG |  |  |  |
| ALP (*G. mellonella*) | Sense | TCGCATAACTCACGCATCTC | RT-qPCR | 104.8 | 0.902 |
|  | Antisense | ATCTTTTGCCTTGGGTTCCT |  |  |  |
| Glycolipid (*G. mellonella*) | Sense | CGCCCGTTATAGATGGAAAA | RT-qPCR | 100.5 | 0.922 |
|  | Antisense | CCTCTACCAATCTCATCAAGCA |  |  |  |
| Prohibitin (*G. mellonella*) | Sense | TGCTCAATTTGATGCTGGAG | RT-qPCR | 101.6 | 0.989 |
|  | Antisense | TTGCCTTTTCAGCTTCCTGT |  |  |  |
| α-amylase (*G. mellonella*) | Sense | CATATGTGGCCTTCCGATCT | RT-qPCR | 99.9 | 0.965 |
|  | Antisense | TTTGAACTCGGTGACAGCAG |  |  |  |
| ADAM metalloprotease (*G. mellonella*) | Sense | CCCGTCATCTCCACCTCTTA | RT-qPCR | 99.6 | 0.925 |
|  | Antisense | CTCGTAGGTTGCAGCACAAA |  |  |  |
| UDP-glucosyltransferase (*G. mellonella*) | Sense | CCCGGATATTGGGCTATTTT | RT-qPCR | 99.8 | 0.915 |
|  | Antisense | GCCGTTGAACACTCCTTCTC |  |  |  |

^a^ underlined sequence indicates *Bam*HI endonuclease site.

^b^ underlined sequence indicates *Hind*III endonuclease site.

^c^ underlined sequence indicates *Sac*I endonuclease site.

NA, not applicable.
